# Toll-like Receptor 3 as a Context-Dependent Molecular Switch in Epithelial Cancers: Balancing Cell Death and Tumor-Supportive Programs

**DOI:** 10.3390/ijms27115075

**Published:** 2026-06-04

**Authors:** Amarilis Pérez-Baños, Andrés Tittarelli

**Affiliations:** Instituto Universitario de Investigación y Desarrollo Tecnológico, Universidad Tecnológica Metropolitana, Santiago 8940577, Chile; amarilisperezbanos@gmail.com

**Keywords:** toll-like receptor 3, regulated cell death, cancer, tumor cell plasticity, signaling pathways

## Abstract

Toll-like receptor 3 (TLR3) is a double-stranded RNA sensor that plays a dual and context-dependent role in epithelial cancers, promoting either regulated cell death (RCD) or tumor-supportive programs. While TLR3 activation has been widely explored for its capacity to induce apoptosis or necroptosis and enhance antitumor immunity, accumulating evidence indicates that cancer cells can use TLR3 signaling to sustain proliferation, migration, stemness, and therapy resistance. In this review, we provide a comprehensive and mechanistic analysis of TLR3 signaling in epithelial cancers, encompassing canonical and non-canonical modules that regulate cancer cell fate. We examine how TLR3 activation engages interconnected RCD programs, including apoptosis, necroptosis, and immunogenic cell death, and contrast these with pathways driving tumor plasticity and progression. Importantly, we discuss the key determinants governing TLR3 signaling output, including signaling complex composition; ligand origin and delivery; TLR3 subcellular localization; and cancer cell-intrinsic factors such as genetic, epigenetic, and metabolic states. We propose an integrative framework in which TLR3 signaling influences cancer cell fate according to cellular and microenvironmental context.

## 1. Introduction

Innate immune sensing pathways are frequently co-opted by cancer cells as components of intrinsic signaling networks to shape disease progression and therapeutic responses [1]. Among pattern recognition receptors (PRRs), toll-like receptors (TLRs) have emerged as critical components of cancer cell-intrinsic signaling, linking cell stress sensing to inflammatory and cell fate-determining pathways [2]. While TLR signaling has been extensively characterized in immune and epithelial cells, its functional relevance in epithelial cancer cells remains incompletely understood and, in many cases, paradoxical [3,4].

TLR3 is a member of the TLR family specialized in the recognition of double-stranded RNA (dsRNA), a molecular pattern associated with viral infections but also endogenously generated under cell stress, cell death, or genomic instability [5,6,7]. Upon ligand binding, TLR3 signals through the adaptor protein TIR-domain-containing adapter-inducing interferon (IFN)-β (TRIF), leading to the activation of transcription factors such as IFN regulatory factor 3 (IRF3) and nuclear factor kappa B (NFκB), and the subsequent induction of type I IFN and pro-inflammatory cytokines [7]. In addition to these canonical pathways, TLR3 can engage signaling modules associated with cell death, including the assembly of receptor-interacting serine/threonine kinase 1 (RIPK1)–fas-associated protein with dead domain (FADD)–caspase-8 complexes, thereby linking extracellular insults and/or cell stress sensing to regulated cell death (RCD) responses [8,9].

In the context of cancer, TLR3 activation has been widely explored as a therapeutic strategy due to its ability to intrinsically induce cancer cell death and enhance, directly or indirectly, antitumor immunity [10,11]. Synthetic dsRNA analogs such as polyinosinic:polycytidylic acid (poly(I:C)) and its derivatives, or dsRNA from oncolytic virus, have shown promising results in preclinical models by promoting cancer cell death and dendritic cell (DC) activation. For instance, poly(I:C)-induced cell death has been reported in multiple epithelial cancer cell models, including prostate [12], breast [13], and lung [14] cancers. Moreover, TLR3 activation has been linked to immunogenic cell death (ICD) features, including the release of danger-associated molecular patterns (DAMPs) and enhanced tumor-associated antigen cross-presentation [15].

Recent advances have redefined cell death as a network of interconnected regulated programs rather than isolated pathways [16]. Within this framework, TLR3 emerges as a key upstream node capable of modulating multiple death and survival outcomes depending on cellular context. Indeed, TLR3 activation can promote cancer cell plasticity, survival, proliferation, migration, and resistance to therapy, often through NFκB-dependent transcriptional programs and pro-inflammatory signaling networks [4,17,18].

Several regulatory determinants shape TLR3 signaling output. Among them, the composition of the signaling platform assembled upon TLR3 activation, including the relative abundance and activity of RIPK1, caspase-8, cellular FLICE-like inhibitory protein (cFLIP), and cellular inhibitor of apoptosis proteins (cIAPs), has been shown to determine whether cancer cells undergo RCD or cell survival [9,14,19,20,21]. Additionally, the nature of the ligand; its mode of delivery; and the cellular context, including oncogenic background and metabolic state, further modulate TLR3 signaling outputs [22,23,24,25]. Finally, the subcellular localization and post-translational modifications of TLR3 may influence ligand accessibility, downstream signaling outputs, and cancer cell responses [26].

Most studies on TLR3 function in cancer cells have focused on individual pathways or specific cellular models, often resulting in partial interpretations that do not fully capture its dual roles. Accordingly, a comprehensive framework explaining how TLR3 integrates diverse signals to determine cell fate in epithelial cancers is still missing. Addressing this gap is essential not only for understanding cancer biology but also for the rational design of TLR3-targeted antitumor therapeutic strategies, which have shown variable efficacy in preclinical and clinical settings [10,11,24]. In this review, we provide a comprehensive and integrative analysis of TLR3 signaling, particularly in epithelial cancer cells, focusing on its dual capacity to promote RCD and tumor progression-associated cell functions. Furthermore, we discuss the key determinants that govern TLR3 functional output, including signaling scaffold composition, ligand context, TLR3 subcellular localization, and tumor microenvironmental cues.

Importantly, because much of the available evidence derives from independent studies performed in different cancer cell models, ligand systems, delivery approaches, and experimental settings, the molecular switch model proposed here should be interpreted as an integrative conceptual framework rather than as a single experimentally validated pathway operating uniformly across epithelial cancers. Throughout the review, we distinguish mechanisms directly supported within defined experimental systems from broader interpretative links that require validation through comparative, time-resolved, and multi-parametric studies.

## 2. Canonical Versus Non-Canonical TLR3 Signaling Pathways in Cancer Cells

TLR3 is unique among TLR family members in that it signals exclusively through the adaptor protein TRIF, thereby defining a myeloid differentiation primary response 88 (MyD88)-independent signaling axis that integrates antiviral responses with cell fate regulation [8]. Upon recognition of dsRNA, TLR3 dimerizes and clusters along the ligand, promoting TRIF recruitment via Toll/IL-1 receptor (TIR) domain interactions [27]. These interactions initiate a modular signaling cascade in which distinct downstream branches are selectively engaged rather than linearly activated: the canonical inflammatory and IFN pathways, as well as non-canonical cell death-associated modules.

To avoid an overly rigid interpretation of TLR3 signaling, in this review we use the terms canonical and non-canonical in an operational rather than absolute sense. Canonical TLR3 signaling refers to the classical TRIF-dependent transcriptional modules that culminate in IRF3- and NFκB-associated gene expression, including type I IFNs, IFN-stimulated genes (ISGs), inflammatory cytokines, and chemokines. By contrast, non-canonical TLR3 signaling refers to outputs that diverge from this classical transcriptional inflammatory/antiviral axis, including TRIF-dependent death-associated complexes as well as TRIF-independent or partially TRIF-independent signaling modules. Thus, some non-canonical outputs may still require TRIF as a scaffold but are considered non-canonical because their primary biological function is not classical IFN/inflammatory gene induction, but rather cell death, migration, plasticity, or rapid transcription-independent signaling.

**Figure 1:**
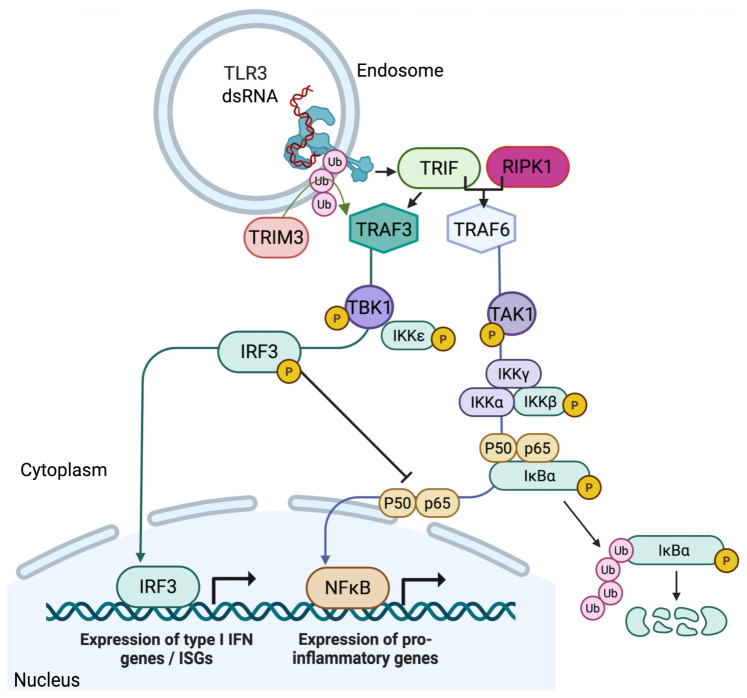
**Canonical TLR3 transcriptional signaling pathways in cancer cells.** Recognition of dsRNA by endosomal TLR3 promotes TRIF-dependent activation of IRF3 and NFκB transcriptional programs through TRAF3/TRAF6-associated signaling complexes. Canonical TLR3 signaling coordinates antiviral and inflammatory responses through TRIF-dependent signaling complexes. Created in BioRender. Tittarelli, A. (2026) https://BioRender.com/rb37fvx.

In the canonical pathway (Figure 1), TRIF functions as a central signaling scaffold that coordinates activation of IRF3 and NFκB. TRIF recruits tumor necrosis factor (TNF) receptor-associated factor (TRAF)3 and TRAF6, which in turn engage downstream kinases such as TANK-binding kinase 1 (TBK1) and IκB kinase (IKK) complexes, leading to IRF3 and NFκB activation, respectively. Activated IRF3 translocates to the nucleus to induce ISG expression, whereas NFκB drives transcription of pro-inflammatory genes [25,28]. Also, canonical TLR3-TBK1-IRF3 signaling is regulated by ubiquitin-dependent mechanisms. In non-small-cell lung carcinoma (NSCLC) models, tripartite motif-containing protein 3 (TRIM3), a RING-domain E3 ubiquitin ligase, physically interacts with TLR3 and catalyzes K63-linked ubiquitination of TLR3 at K808 [29]. This modification enhances TLR3 cleavage (required for its functional activation), TBK1 and IRF3 phosphorylation, and IFN-β secretion upon poly(I:C) stimulation, positioning TRIM3 as a positive regulator of the canonical IFN-producing arm of TLR3 signaling. Additionally, IRF3 can directly modulate NFκB activity by limiting p65 nuclear translocation, thereby constraining excessive inflammatory responses [30]. This highlights that canonical TLR3 signaling is not a simple bifurcation but rather a dynamically regulated network in which antiviral and inflammatory outputs are tightly coordinated.

Moreover, TLR3 activation promotes the formation of higher-order signaling platforms, collectively referred to as the “TRIFosome”, that function as integrative hubs which serve as platforms for signal amplification and integration. Indeed, recent work demonstrates that TRIF can oligomerize into filamentous structures that recruit key signaling intermediates such as RIPK1 and RIPK3, which is promoted and controlled by the nucleic acid sensor Z-DNA-binding protein 1 (ZBP1) [31,32]. Importantly, TRIFosome formation is temporally regulated, occurring after initial signaling events and correlating with sustained NFκB activation. This suggests a two-phase model in which early signaling is mediated by monomeric TRIF, while later responses depend on the formation of a dynamic and stable signaling platform capable of integrating multiple inputs to determine downstream cellular responses.

In addition to canonical transcriptional responses, TLR3 signaling engages non-canonical pathways that directly regulate cell fate. Central to this process is the ability of TRIF to interact with receptor-interacting protein kinases through its RIP homotypic interaction motif (RHIM) domain. Early studies in human embryonic kidney cells demonstrated that TRIF could induce apoptosis or necroptosis independently of IFN or NFκB signaling through the recruitment of RIPK1 and the activation of the FADD–caspase-8 axis [8]. Indeed, RIPK1 functions as a scaffold protein that can promote NFκB activation or, alternatively, participate in the formation of pro-death complexes depending on its interaction context and post-translational modifications [31]. This dual role positions RIPK1 as a critical node linking canonical and non-canonical TLR3 signaling.

**Figure 2:**
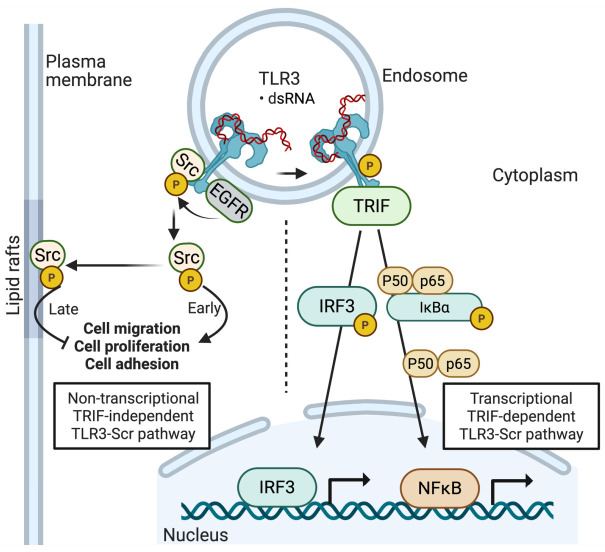
**Non-canonical TLR3 signaling pathways linking dsRNA sensing to Src/EGFR-dependent cancer cell plasticity.** In addition to canonical TRIF-dependent transcriptional signaling, TLR3 can engage non-canonical pathways involving Src family kinases and EGFR. These signaling modules rapidly modulate migratory and proliferative cellular responses independently of classical IRF3/NFκB transcriptional activation, linking innate immune sensing to oncogenic signaling networks. Created in BioRender. Tittarelli, A. (2026) https://BioRender.com/19lb8zw.

A TRIF-independent branch of TLR3 signaling mediated by the proto-oncogene Src has been identified in nontumor cell models as an additional non-canonical TLR3 signaling pathway. This pathway does not require gene transcriptional induction and rapidly modulates cellular functions such as cell migration, adhesion, and proliferation [33]. The authors determined that these cell responses are biphasic, with an early increase in motility followed by sustained inhibition due to sequestration of activated Src into lipid rafts. Importantly, this pathway operates independently of all the canonical TLR3 signaling components, including TRIF, IRF3, and NFκB, highlighting a direct link between innate immune sensing and oncogenic signaling networks. Complementing this mechanism, a parallel work from the same group demonstrated that TLR3 signaling is tightly coupled to receptor tyrosine kinase activity in fibrosarcoma cells, particularly through the epidermal growth factor receptor (EGFR). Upon dsRNA stimulation, EGFR is recruited to TLR3 in endosomal compartments and functions upstream of Src, enabling the phosphorylation of critical tyrosine residues in the cytoplasmic domain of TLR3 that are required for downstream TRIF recruitment and transcriptional signaling [34] (Figure 2). In the absence of EGFR activity, TLR3 fails to induce IRF3 activation and antiviral gene expression, establishing EGFR as an essential component of the TLR3 signaling machinery. These findings link TLR3 signaling to growth factor-dependent pathways regulating cell plasticity.

Taken together, recent advances support a revised model of TLR3 signaling as a modular and temporally organized process. Early signaling events are mediated by monomeric TRIF complexes that rapidly activate IFN responses, while subsequent assembly of TRIFosomes enables sustained NFκB activation and integration with cell death pathways. The recruitment of additional regulators such as ZBP1 further refines these outputs by modulating the composition and kinetics of signaling complexes. This framework provides a mechanistic basis for understanding how TLR3 can generate diverse and sometimes opposing cellular outcomes (Table 1). Rather than representing distinct pathways, canonical and non-canonical signaling are highly interconnected.

## 3. TLR3-Driven Cancer Cell Death Pathways

Beyond its canonical antiviral and inflammatory functions, TLR3 can engage regulated cell death programs when cancer cells retain sufficient death-signaling competence. These outcomes should not be interpreted as linear consequences of dsRNA sensing. Rather, TLR3-driven RCD emerges when ligand sensing, TRIF platform assembly, RIPK1/RIPK3 availability, caspase-8 activity, and inhibitory checkpoints converge to lower the death threshold of cancer cells.

### 3.1. TLR3 Activation Promotes Apoptosis in Cancer Cells

In apoptosis-permissive cancer contexts, TLR3 can function as a conditional non-canonical death receptor, whose apoptotic activity is actively regulated rather than intrinsically programmed. Unlike other TLRs, TLR3 possesses the intrinsic capacity to couple dsRNA recognition to caspase-dependent cell death through TRIF complexes with procaspase-8, FADD, and RIPK1, functioning independently of classical death receptors such as Fas or TNF-related apoptosis-inducing ligand (TRAIL) receptors [8,14]. TLR3-mediated apoptosis has been documented in multiple epithelial tumor models, although the magnitude and signaling requirements vary substantially between cancer types. Early studies investigating TLR3-driven apoptosis in cancer cells showed that in apoptosis-competent breast cancer cell lines, poly(I:C) induces apoptosis in a TLR3-, TRIF-, and interleukin (IL)-1 receptor-associated kinase-4 (IRAK-4)-dependent manner [13,35]. Remarkably, the authors showed that in these breast cancer models, poly(I:C)-induced apoptosis required caspase-8/-3 activation, NFκB and type I IFN signaling, although IFN alone was insufficient to drive cancer cell death. Consistent with this, in ovarian cancer cell models retaining intact TBK1/IRF3 signaling, viral dsRNA stimulation can promote TLR3-mediated apoptosis [36].

Notably, the requirement for sensitizing conditions reveals the existence of intrinsic inhibitory thresholds that actively constrain TLR3-driven apoptosis. In cervical cancer cells, the inhibition of protein synthesis renders cells susceptible to poly(I:C)-induced apoptosis, suggesting that short-lived antiapoptotic proteins actively restrain cell death signaling [37]. Similarly, pharmacological priming of breast cancer cells with retinoic acid (RA) amplifies TLR3-driven apoptosis through a type I IFN-dependent autocrine loop that induces TRAIL and its receptors [38,39]. Additionally, regulation of TLR3 trafficking and signaling competence has been implicated in sensitization of cancer cell to apoptosis. In paclitaxel-resistant colon cancer cells, poly(I:C) induces upregulation of the chaperone UNC93B1, promoting TLR3 trafficking to endosomes and enhancing its functional activation [40]. This results in robust IFN-β production, which acts in an autocrine manner to promote apoptosis. Additionally, cytokine-mediated priming further contributes to the establishment of an apoptosis-permissive state in cancer cells. For example, in prostate cancer models, IL-24 sensitizes cells to TLR3-dependent apoptosis by remodeling the composition of its signaling scaffold complex, reducing cFLIP incorporation and facilitating caspase-8 activation [41]. Similarly, IL-27 enhances TLR3 expression and reinforces IFN-β-dependent autocrine signaling, thereby increasing apoptotic responses in prostate cancer cell lines [42]. It is worth noting that tumor levels of both cytokines, IL-24 and IL-27, have been implicated in promoting antitumor immunity and correlated with good clinical prognosis in patients [43,44]. Altogether, these findings indicate that TLR3-dependent apoptotic competence in cancer cells could be modulated by the tumor cytokine milieu and the regulatory state of its signaling platform.

At the transcriptional level, NFκB has also been implicated as a critical survival checkpoint downstream of TLR3. In opposition to the NFκB p65-dependent proapoptotic signaling observed in poly(I:C)-treated breast cancer cells [13], in head and neck squamous cell carcinoma (HNSCC), defective NFκB activation correlates with increased susceptibility to poly(I:C)-induced apoptosis, whereas restoration of NFκB signaling reduces cell death [45]. These observations indicate that NFκB functions as a bifunctional regulator whose output depends on the balance between pro-survival and pro-death transcriptional programs. While NFκB can promote the expression of pro-survival genes, including antiapoptotic factors such as cFLIP and cIAPs, it can also cooperate with IRF3 and type I IFN signaling to facilitate apoptotic programs under specific conditions. Thus, the functional outcome of NFκB activation appears to depend on the balance between its transcriptional targets, the timing of its activation, and its integration with parallel signaling pathways. In line with this, RIPK1 plays a central scaffold role in balancing survival and apoptotic signaling, as reduced RIPK1 expression in metastatic HNSCC cells impairs NFκB activation while preserving IRF3 signaling, resulting in enhanced apoptosis [46]. Thus, NFκB and RIPK1 may either restrain or facilitate apoptosis depending on signaling context.

Additional signaling modules can also contribute to TLR3-driven apoptosis in a context-dependent manner. In prostate cancer cells, TLR3 activation engages stress-associated mitogen-activated protein kinases (MAPKs), including p38 and c-JUN N-terminal kinase (JNK), in a protein kinase C (PKC)α-dependent manner, which is required for caspase activation and apoptotic execution [12]. In addition, metabolic and survival pathways further modulate apoptotic susceptibility to TLR3 activation, as it suppresses phosphoinositide 3-kinase (PI3K)/Akt signaling and induces coordinated changes in cell cycle regulators, while autophagy acts as a cytoprotective mechanism that limits apoptosis in prostate cancer cells [47]. Consistently, autophagy-dependent degradation of TRIF has been shown to restrain apoptotic signaling in hepatocellular carcinoma (HCC) [48]. Indeed, TLR3 expression is frequently downregulated in HCC, and this loss correlates with reduced apoptosis, enhanced tumor progression, and poor patient prognosis, whereas restoration of TLR3 expression re-sensitizes HCC cells to apoptosis, indicating that TLR3 downregulation may represent a tumor-intrinsic escape mechanism [49].

A final layer of apoptotic regulation is imposed by inhibitory checkpoint modules that control whether TRIF-associated complexes progress toward caspase activation. For example, in epithelial keratinocytes, caspase-8 activation by TLR3 ligands is tightly controlled by cIAPs [50]. These regulatory elements converge in a multiprotein platform termed the “ripoptosome”, a RIPK1–FADD–caspase-8-containing complex that integrates apoptotic and necroptotic signals. In squamous cell carcinoma (SCC) cells, TLR3 activation promotes TRIF recruitment to this complex, while loss of cIAPs facilitates ripoptosome assembly and enables the activation of either apoptosis or necroptosis depending on caspase activity and cFLIP isoform composition [19]. Similarly, cFLIP and cIAPs function as a coordinated inhibitory network that defines the apoptotic threshold in NSCLC cells, as the inhibition of either factor sensitizes cancer cells to TLR3-mediated apoptosis, whereas non-transformed cells require simultaneous release of both inhibitory mechanisms [20]. In line with this model, chemotherapeutic agents such as cisplatin can lower this threshold by downregulating cFLIP, thereby restoring caspase-8 activation and apoptosis in malignant mesothelioma cells [51].

**Figure 3:**
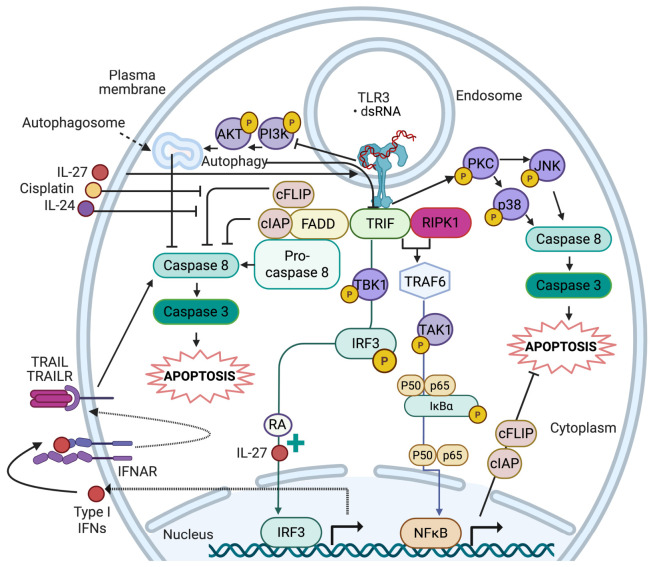
**Context-dependent regulation of TLR3-driven apoptosis in cancer cells through integration of death signaling, intracellular checkpoints, and sensitizing pathways.** TLR3 activation promotes the assembly of TRIF-associated death signaling complexes containing RIPK1, FADD, and caspase-8. The execution of apoptosis is dynamically regulated by intracellular checkpoints including cFLIP, cIAPs, autophagy, NFκB signaling, cytokine-mediated sensitization, and IFN-dependent amplification loops, collectively determining apoptotic competence downstream of TLR3. Created in BioRender. Tittarelli, A. (2026) https://BioRender.com/icfl8zn.

Collectively, these findings suggest that TLR3-mediated apoptosis depends on death-complex assembly and inhibitory checkpoint regulation (Figure 3). Apoptotic execution depends on the assembly of TRIF-associated death complexes, the availability of caspase-8, and the balance between inhibitory checkpoints such as cFLIP, cIAPs, autophagy, and NFκB-driven survival programs. In this framework, TLR3 acts as a conditional death-inducing platform whose apoptotic output is shaped by cancer cell state, cytokine milieu, and therapeutic perturbations. This explains why TLR3 activation induces robust apoptosis in some tumor models but fails to do so in others.

### 3.2. TLR3-Driven Necroptosis and ICD as Interconnected RCD Outputs

Necroptosis, a form of regulated necrotic cell death mediated by RIPK1, RIPK3 and mixed lineage kinase domain-like protein (MLKL) [52], represents an alternative TLR3-driven RCD output that becomes accessible when the apoptotic checkpoint architecture is altered. Rather than operating as an independent pathway, TLR3-induced necroptosis emerges from the same TRIF/RIPK1 signaling axis that can also support apoptosis, with the outcome depending on RIPK3/MLKL availability, caspase-8 activity, and cIAP/cFLIP-mediated inhibition. Seminal studies performed in murine macrophages and fibroblasts demonstrated that upon activation, TLR3 engages the adaptor TRIF, which interacts with RIPK3 and triggers MLKL-mediated membrane permeabilization independently of classical death receptor pathways [53]. However, this pathway is actively restrained by inhibitory checkpoints, positioning necroptosis as a regulated rather than default outcome of TLR3 activation. Also, RIPK3 expression is frequently lost in several epithelial tumors, restricting necroptotic competence. RIPK1 exerts a critical suppressive role by recruiting inhibitory complexes involving caspase-8 and cFLIP, thereby restraining RIPK3 activation. Disruption of this checkpoint, such as through caspase inhibition or altered RIPK1 function, enables TRIF-dependent necroptotic signaling [54].

In cancer cell models, the execution of necroptosis downstream of TLR3 can act as a direct antitumor mechanism and depends on both intracellular signaling thresholds and environmental context. In colorectal cancer cells, poly(I:C) stimulation in combination with transforming growth factor (TGF)-β-activated kinase 1 (TAK1) inhibition induces an initial necroptotic response followed by RIPK1-dependent apoptosis, highlighting the dynamic interplay between both RCD pathways [55]. Similarly, TLR3 activation coupled to caspase inhibition triggers RIPK1/RIPK3-dependent necroptosis independently of TNF or type I IFN signaling and is associated with reactive oxygen species (ROS) production and colon carcinoma regression in vivo [56]. Importantly, the susceptibility of cancer cells to TLR3-induced necroptosis is also regulated at the level of TLR3 expression and inflammatory priming. In cholangiocarcinoma models, IFN-γ induces TLR3 expression through IRF1-dependent mechanisms and sensitizes cancer cells to poly(I:C)-induced death [57]. Under conditions where cIAPs are antagonized and caspase activity is inhibited, this IFN-γ-mediated priming enables robust activation of the RIPK1-RIPK3-MLKL axis, leading to necroptosis accompanied by DAMP release and DC activation, indicative of ICD.

Importantly, the transition from TLR3-driven cytotoxicity to ICD depends not only on the modality of cell death, but also on whether dying tumor cells generate immunostimulatory signals such as type I IFNs, DAMPs, endogenous dsRNA, and inflammatory cytokines. Therefore, TLR3-driven RCD should be interpreted not only by its execution mechanism, but also by its capacity to remodel antitumor immunity.

Indeed, several lines of research have shown that TLR3 activation in cancer cells can promote ICD. Chemotherapy-induced stress promotes the release of self-derived RNA by cancer cells, which is sensed in an autocrine manner by TLR3, triggering a type I IFN response essential for antitumor immunity [15]. Extending this concept, epigenetic dysregulation can also serve as a source of immunostimulatory dsRNA. Inhibition of DNA methylation leads to the transcriptional activation of endogenous retroviruses, generating dsRNA species that mimic viral infection and engage dsRNA sensors, including TLR3 and melanoma differentiation-associated protein 5 (MDA5) [58]. This activation induces IFN signaling, ISG expression, and apoptotic responses in ovarian cancer cells, linking epigenetic reprogramming to innate immune activation. Notably, TLR3 contributes functionally to this response, as its knockdown significantly reduces IFN induction. Moreover, intron-derived dsRNA generated from repetitive elements can also activate TLR3-dependent immune surveillance programs in pancreatic cancer cell models, although this pathway is actively suppressed by MYC/MIZ1-mediated control of vesicular trafficking [24]. Together, these findings support a model in which endogenous dsRNA, generated either by therapy-induced damage or epigenetic derepression, acts as a central trigger of TLR3-mediated ICD programs in cancer cells.

TLR3-dependent IFN-β signaling can also remodel the immune composition of the tumor microenvironment (TME) toward an antitumor state. In NSCLC models, activation of the TRIM3/TLR3 axis sustains IFN-β secretion and suppresses tumor progression while increasing CD4^+^ T, natural killer (NK) cell, and M1 macrophage infiltration and reducing M2 macrophage abundance in tumors [29]. At the regulatory level, RIPK1 emerges as a central checkpoint controlling the transition from inflammatory signaling to ICD. Recent evidence demonstrates that pharmacological degradation of RIPK1 unleashes necroptosis and amplifies NFκB and IFN signaling, thereby converting tumor cells into potent sources of immunogenic stimuli that enhance antitumor immunity [59]. These findings suggest that RIPK1 functions as a gatekeeper that restrains TLR3-driven ICD by limiting RIPK3 activation and downstream immune activation.

In addition to IFN-driven immunogenicity, TLR3 activation has also been linked to alternative RCD programs, including ferroptosis, further expanding the spectrum of TLR3-mediated cytotoxic and immunomodulatory effects. In HCC models, poly(I:C) enhances the abscopal effect of radiotherapy by promoting ferroptosis in both irradiated and distant tumors [60]. This effect depends on DC activation and subsequent CD8^+^ T cell responses, inducing lipid peroxidation and ferroptotic cancer cell death.

Nevertheless, it has been observed that TLR3-induced cell death remains restricted to apoptosis, even under conditions that might favor necroptosis. In NSCLC and other epithelial tumors, poly(I:C)-induced cell death is strictly caspase-dependent, as caspase inhibition abrogates cell death while inhibition of RIPK1 or RIPK3 has no protective effect [14,20]. Similarly, in melanoma and prostate cancer cells that naturally do not express RIPK3, TLR3-driven cell death remains insensitive to RIPK1 inhibition, reflecting the absence of necroptotic signaling [41]. These findings emphasize that TLR3-induced necroptosis requires a permissive molecular context and may frequently be suppressed in epithelial cancers.

**Figure 4:**
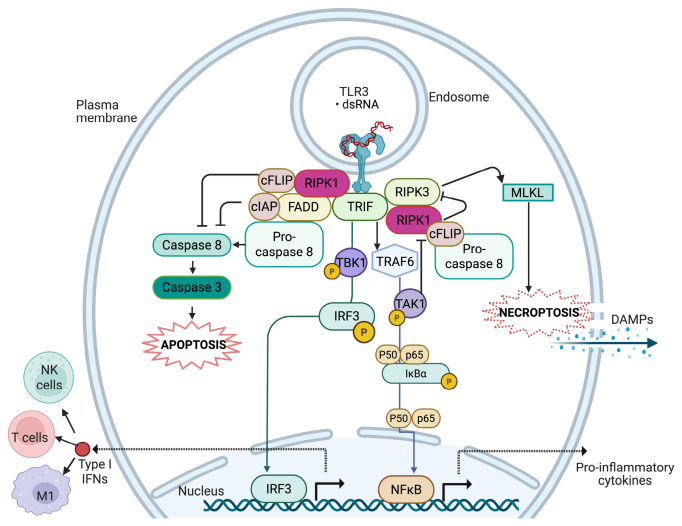
**TLR3 as a context-dependent hub integrating apoptosis, necroptosis, and immunogenic cell death in cancer cells.** TLR3 signaling engages interconnected RCD programs whose execution depends on intracellular signaling thresholds and cancer cell state. Depending on RIPK3 competence, caspase activity, and inflammatory signaling context, TLR3 activation can promote apoptosis, necroptosis, and ICD, linking innate immune sensing with the immunogenic quality of tumor cell death. Created in BioRender. Tittarelli, A. (2026) https://BioRender.com/1sfdpaj.

Taken together, these observations support the notion that TLR3 signaling does not engage a single predefined RCD pathway but rather operates within a dynamic and interconnected network of RCD programs (Figure 4). Importantly, the ability of TLR3 to induce apoptosis, necroptosis, or ICD is highly heterogeneous across epithelial cancers and appears to depend on preserved death-signaling competence, including RIPK3 expression, caspase-8 activity, endosomal trafficking, and permissive inflammatory signaling states. Thus, apoptosis, necroptosis, and ICD are not isolated consequences of TLR3 activation, but alternative outputs of a shared signaling continuum controlled by death competence and immune-stimulatory thresholds.

## 4. Tumor-Promoting Activities of Cancer Cell TLR3

In contrast to the TLR3-driven RCD programs elicited in cancer cells, accumulating evidence indicates that TLR3 signaling can be functionally reprogrammed by cancer cells to support tumor-promoting outputs. Although substantial evidence supports tumor-promoting functions of TLR3 in specific epithelial cancer contexts, these programs frequently emerge in apoptosis-resistant, inflammatory, therapy-adapted, or oncogenically rewired cellular states. In multiple cancer cell types, constitutive or therapy-induced activation of TLR3 drives proliferation, migration, metabolic adaptation, stemness, and the establishment of pro-inflammatory circuits that support tumor progression. Importantly, many of these tumor-promoting outputs have been described under highly specific experimental and molecular contexts, suggesting that these effects likely depend on pre-existing tumor plasticity programs. The following sections summarize the major tumor-promoting activities associated with cancer cell-intrinsic TLR3 signaling.

### 4.1. Cancer Cell Proliferation and Survival Pathways Activated by TLR3 Signaling

In several epithelial cancer models, including melanoma, pancreatic, and breast cancer, high basal TLR3 activity sustains inflammatory survival programs characterized by IL-6/signal transducer and activator of transcription 3 (STAT3) signaling, IFN-β production, and Wnt5a-associated migratory phenotypes [61,62]. Functional inhibition of TLR3 reduces STAT3 activation, proliferation, migration, and therapy resistance, indicating that constitutive TLR3 signaling can support adaptive tumor cell fitness. In this context, chemotherapy-induced cellular stress can paradoxically activate tumor-intrinsic innate immune pathways that promote survival rather than cell death. In HNSCC, cisplatin induces the release of DAMPs that activate TLR3 signaling, leading to the upregulation of IFN-β and C-C motif chemokine ligand (CCL)5. This response enhances tumor cell survival and contributes to chemoresistance, as TLR3 knockdown increases cisplatin sensitivity and reduces tumor growth in vivo [63]. These findings position TLR3 as a sensor of therapy-induced damage that can drive adaptive resistance mechanisms in cancer cells.

TLR3 signaling can also engage hypoxia-associated adaptive programs in apoptosis-resistant epithelial cancers. In prostate and breast cancer models, poly(I:C)-mediated TLR3 activation promotes NFκB/extracellular signal-regulated kinase (ERK)-dependent hypoxia-induced factor-1α (HIF-1α) accumulation under normoxic conditions, inducing vascular endothelial growth factor (VEGF) expression, antiapoptotic signaling, vasculogenic mimicry, and resistance to apoptosis [64,65]. Importantly, pharmacological or genetic inhibition of HIF-1α restores susceptibility to TLR3-mediated cell death, highlighting hypoxia-associated transcriptional programs as critical regulators of TLR3 functional output.

Finally, it has been reported that TLR3 activity can be directly reprogrammed by oncogenic drivers to support tumor progression in NSCLC. The human papillomavirus E6 oncoprotein induces a marked upregulation of TLR3 expression, which becomes functionally required for the acquisition of proliferative and invasive phenotypes [66]. Silencing of TLR3 effectively abrogates E6-driven tumor cell bioactivities, demonstrating a causal role for TLR3 in this process. Mechanistically, this effect is mediated through activation of a non-canonical signaling axis involving Src phosphorylation, while remaining largely independent of the classical TRIF pathway. These findings reveal that TLR3 can support oncogenic signaling programs when co-opted by viral oncoproteins, shifting its role from antiviral defense to the promotion of tumor growth and invasion.

**Figure 5:**
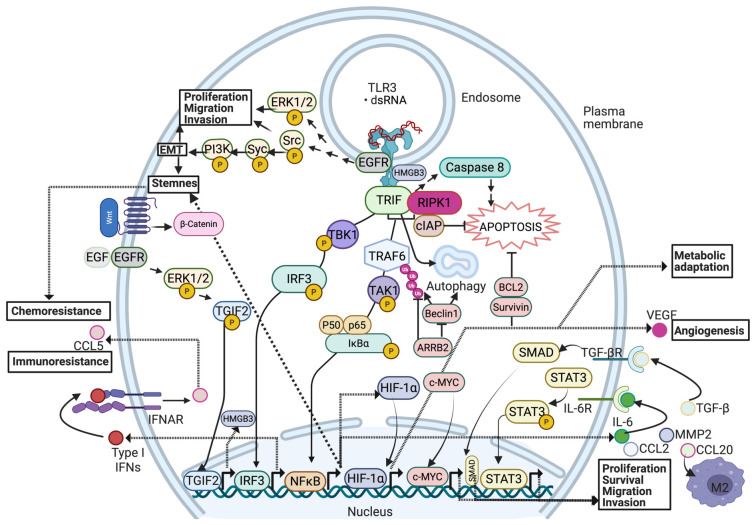
**TLR3-driven signaling networks promoting epithelial cancer cell plasticity, survival, and tumor progression.** Under specific oncogenic, inflammatory, metabolic, and therapy-adapted states, TLR3 signaling can be redirected toward tumor-supportive outputs. These include activation of NFκB-, STAT3-, HIF-1α-, EGFR/Src-, and TGF-β-associated signaling programs linked to proliferation, epithelial plasticity, stemness, metabolic adaptation, immune evasion, and therapy resistance. Created in BioRender. Tittarelli, A. (2026) https://BioRender.com/6ywyhe9.

Taken together, this evidence indicates that TLR3 signaling can sustain cancer cell proliferation and survival through multiple, non-mutually exclusive mechanisms that extend beyond its canonical antiviral role (Figure 5). Basal TLR3 activity can support IL-6-STAT3-dependent growth, while therapy-induced activation reinforces adaptive resistance programs, and oncogenic drivers can redirect TLR3 signaling toward non-canonical pathways that favor proliferation and invasion.

### 4.2. Migration and Epithelial-to-Mesenchymal Transition (EMT)-like Programs Driven by TLR3 Signaling

In melanoma and pancreatic cancer cells, constitutive TLR3 activity is associated with increased expression of the non-canonical Wnt ligand Wnt5a, a key regulator of cell motility. This axis is mediated through IL-6-dependent STAT3 activation, establishing a TLR3-IL-6-STAT3-Wnt5a signaling cascade that promotes cancer cell migration [61]. Similarly, in lung cancer cell models, poly(I:C)-mediated TLR3 activation enhances migration, invasion, and colony formation through NFκB activation and production of protumorigenic factors such as IL-6, CCL2, and matrix metalloproteinase (MMP)2 [67]. Mechanistically, this response depends on a cyclic adenosine monophosphate (cAMP)–AMP-activated protein kinase (AMPK)–TAK1 signaling axis upstream of NFκB, highlighting how inflammatory signaling networks downstream of TLR3 can directly drive tumor progression.

TLR3-driven migration is tightly regulated by intracellular checkpoints that determine whether signaling promotes invasion or cell death. In cholangiocarcinoma models, TLR3 activation enhances cancer cell invasion through the NFκB and MAPK pathways, whereas loss of RIPK1 further exacerbates this invasive phenotype [68]. Conversely, restoration of death signaling through cIAPs antagonism shifts TLR3 responses toward apoptosis and necroptosis and suppresses invasion, highlighting RIPK1 as an important regulator of TLR3 functional output.

TLR3 can also cooperate with growth factor signaling networks to promote tumor cell plasticity. In prostate cancer models, high basal TLR3 expression is associated with therapy-resistant cells and promotes motility, migration, invasion, and intra-abdominal metastatic dissemination in an orthotopic xenograft model [69]. Transcriptomic analyses revealed enrichment of migration- and invasion-related programs, with EGFR activity contributing to the migratory phenotype. Notably, exogenous activation of TLR3 with poly(I:C) in these cells triggers caspase-3/7 activation and poly(ADP-ribose) polymerase (PARP) cleavage, revealing that the same receptor can sustain invasive traits when basally active but become an apoptotic vulnerability upon ligand engagement. In line with this concept, exosomal RNA derived from human immunodeficiency virus-infected T cells activates TLR3 in recipient cancer cells in an EGFR-dependent manner, leading to ERK1/2 phosphorylation and enhanced proliferation, migration, and invasion [70]. Importantly, both EGFR signaling and TLR3 engagement are required for this effect, establishing a paracrine TLR3-EGFR-ERK signaling axis that promotes tumor progression.

Autophagy has also emerged as an important regulatory component of TLR3 signaling in cancer cells. In lung and prostate cancer models, TLR3-induced autophagy sustains TRAF6/NFκB signaling, cytokine production, and invasive behavior, whereas inhibition of autophagy or disruption of TRAF6 regulatory interactions suppresses migration and tumor-promoting inflammatory responses [71,72,73]. These findings suggest that autophagy can reinforce protumoral TLR3 signaling by stabilizing inflammatory and adaptive signaling networks.

Emerging evidence indicates that TLR3 signaling can engage epithelial plasticity programs in several epithelial cancer contexts. In keratinocytes, ovarian cancer, and ESCC models, TLR3 activation has been associated with EMT-like phenotypes, migratory behavior, and activation of the Src/Syk-, PI3K-, NFκB-, and TGF-β-associated signaling pathways [74,75,76].

Finally, TLR3 signaling can be co-opted by oncogenic programs to sustain tumor progression. In esophageal squamous cell carcinoma (ESCC), the TGF-β-induced factor homeobox 2 (TGIF2)–high mobility group box (HMGB)3 axis interacts with TLR3 to promote NFκB activation and TGF-β/SMAD signaling, driving proliferation, invasion, and metastasis both in vitro and in vivo [77]. Notably, disruption of TGF-β signaling abrogates these effects, indicating that TLR3 can function as an upstream regulator of protumoral plasticity programs. Collectively, these findings suggest that in certain epithelial tumor contexts, TLR3 signaling has been associated with epithelial plasticity programs, integrating inflammatory, metabolic, and oncogenic cues to coordinate EMT-like programs and invasive behavior.

### 4.3. TLR3 Activation Induces Cancer Cell Stemness

Beyond enabling motility, EMT programs can confer progenitor-like features, allowing cells to acquire stem-like phenotypes in response to environmental and intracellular cues [77]. In cancer, this plasticity is closely linked to the emergence of cancer stem cells (CSCs), a subpopulation with enhanced tumor-initiating capacity, resistance to therapy, and ability to sustain tumor heterogeneity [78]. In this context, emerging evidence indicates that TLR3 signaling can promote the acquisition of stem-like states in cancer cells. These effects appear particularly prominent in breast and colorectal cancer models exhibiting inflammatory and Wnt-associated signaling states.

TLR3-associated stemness programs are closely linked to metabolic adaptation and inflammatory signaling. In breast, colorectal, HNSCC, and pharyngeal cancer models, TLR3 activation promotes CSC-associated phenotypes together with glycolytic rewiring, HIF-1α accumulation, NFκB activation, and MAPK/Myc-dependent metabolic adaptation [17,79,80,81,82]. These coordinated changes enhance tumorsphere formation, therapy resistance, migratory behavior, and adaptation to microenvironmental stress, suggesting that TLR3 signaling can reinforce interconnected plasticity programs rather than isolated stemness-associated traits.

Collectively, these findings indicate that TLR3 signaling functions as a central regulator of cancer cell plasticity by coupling EMT-associated programs with stemness, metabolic adaptation, and therapy resistance. Rather than acting as a linear immune sensor, TLR3 integrates inflammatory signaling, transcriptional reprogramming, and metabolic rewiring to enable the dynamic interconversion of cancer cell states. This coordinated network promotes the emergence and maintenance of CSC populations, thereby sustaining tumor heterogeneity, adaptive fitness, and long-term disease progression.

### 4.4. Cancer Cell TLR3 Signaling Induces Pro-Inflammatory Circuits That Support Tumor Progression

TLR3 activation in cancer cells not only regulates intrinsic cancer cell behavior but also plays a central role in shaping the inflammatory landscape of the TME. Rather than inducing a uniform immune response, TLR3 signaling generates functionally divergent inflammatory circuits that can either constrain or support tumor progression. In melanoma, glioma, and breast cancer cells, stimulation with poly(A:U) and type I IFN induces the secretion of C-X-C motif chemokine ligand (CXCL)10 and CCL5, which exert opposing effects on antitumor immune response [83]. While CXCL10 promotes the recruitment of CXCR3^+^ effector CD8^+^ T cells and is required for antitumor responses, CCL5 signaling through CCR5 limits immunotherapeutic efficacy. Accordingly, blockade of CCL5 or CCR5 enhances tumor control, whereas disruption of CXCR3 signaling abolishes the therapeutic benefit, demonstrating that TLR3-driven chemokine networks can be selectively modulated to optimize antitumor immune responses. Similarly, TLR3-driven inflammatory signaling can promote the recruitment and polarization of tumor-supportive immune populations. In prostate cancer, TLR3-dependent autophagy induces the secretion of CCL20, which recruits macrophages and promotes their polarization toward an M2 phenotype, reinforcing tumor progression [73]. In addition to immune cell recruitment, TLR3 signaling can activate protumoral inflammatory circuits that reinforce tumor cell survival and progression. In lung cancer cells, poly(I:C)-induced TLR3 activation promotes IL-6 secretion and subsequent autocrine activation of the JAK2/STAT3 pathway, which supports survival and metastatic traits [84]. Importantly, inhibition of this axis enhances TLR3-mediated apoptosis, highlighting the existence of cytokine-driven feedback loops that counteract cell death signaling.

Collectively, these data establish TLR3 signaling as a central regulator of tumor cell plasticity that operates across multiple interconnected biological scales. Beyond its canonical role in innate immune sensing, TLR3 integrates inflammatory signaling, transcriptional reprogramming, metabolic adaptation, and microenvironmental interactions to coordinate cancer cell proliferation, survival, invasion, and stemness (Figure 5). At the cellular level, tumor-supportive TLR3 outputs appear enriched in cancer models exhibiting oncogenic rewiring, chronic inflammatory adaptation, hypoxia-associated signaling, or EMT/stemness-associated plasticity. In parallel, TLR3-driven signaling extends beyond cancer cells to shape the TME by orchestrating pro-inflammatory circuits that recruit and polarize immune populations toward tumor-supportive phenotypes. Importantly, these processes are not independent but form a highly interconnected network of feedback loops in which inflammatory, metabolic, and stemness-associated pathways reinforce one another.

## 5. Regulatory Determinants of TLR3 Signaling Plasticity in Cancer Cells

Importantly, the apparently contradictory outcomes associated with TLR3 signaling across epithelial cancer models likely reflect differences in cellular and signaling context rather than fundamentally distinct receptor functions. However, because most available evidence derives from independent studies performed in different tumor models and experimental conditions, these observations should not be interpreted as definitive cancer-type-specific TLR3 programs. Instead, current data suggest that factors such as RIPK1/RIPK3/caspase-8 competence, cFLIP/cIAP-dependent inhibitory thresholds, p53 status, HIF-1α activity, autophagy, inflammatory adaptation, and oncogenic rewiring may influence TLR3 signaling output in a context-dependent manner. Accordingly, TLR3 activation has been associated with apoptosis, necroptosis, or ICD in some experimental systems, while in others it correlates with migration, invasion, stemness, metabolic adaptation, chemoresistance, or tumor-supportive inflammatory signaling. These context-dependent outcomes are summarized in Table 2 and integrated into the molecular switch model proposed in this review.

Despite the identification of multiple determinants regulating TLR3 signaling, a key challenge remains the integration of these variables into a unified mechanistic framework. Based on the evidence discussed above, we propose that TLR3 signaling output in cancer cells emerges from the dynamic interplay between three principal regulatory axes: (i) spatial regulation, (ii) ligand routing and delivery-dependent signaling, and (iii) cell-intrinsic checkpoints.

### 5.1. Spatial Regulation of TLR3 Signaling

Emerging evidence indicates that TLR3 signaling is critically shaped by its subcellular localization, which determines distinct functional outcomes in cancer cells. In breast cancer cells, plasma membrane-associated TLR3 engages a MyD88-dependent signaling cascade involving IRAK1, TRAF6, and TAK1, leading to NFκB activation, IL-6 production, and cyclin D1 upregulation, thereby promoting proliferation [85]. Post-translational modifications further fine-tune TLR3 subcellular localization and signaling. In pancreatic cancer models, chemotherapeutic stress induces JAK1-dependent phosphorylation of TLR3 at serine 155, enabling its nuclear translocation via importin α5 [26]. Nuclear TLR3 engages oncogenic signaling pathways rather than canonical antiviral responses, illustrating how spatial redistribution can fundamentally alter TLR3 function.

The endoplasmic reticulum (ER)-resident chaperone UNC93B1 adds an additional layer to the spatial control of TLR3 signaling. Although UNC93B1 has classically been viewed as a trafficking factor that delivers nucleic acid-sensing TLRs from the ER to endosomal compartments, recent evidence indicates that its function extends beyond receptor transport. UNC93B1 stabilizes TLR3 at the protein level, thereby preserving the pool of receptors available for subsequent endosomal routing and activation. Thus, UNC93B1 acts upstream of ligand sensing by determining whether TLR3 is maintained as a signaling-competent receptor [86]. This distinction is particularly relevant for cancer cells, where the abundance and localization of TLR3 may determine whether dsRNA sensing reaches a pro-death threshold. In paclitaxel-resistant colon cancer cells, poly(I:C) combined with paclitaxel upregulates both TLR3 and UNC93B1, enhancing IFN-β secretion and promoting apoptosis through a TLR3-UNC93B1-IFN-β signaling axis. These findings suggest that UNC93B1-dependent stabilization and routing of TLR3 may facilitate an endosomal signaling state permissive for IFN-associated apoptotic responses [40]. Importantly, UNC93B1 may also be functionally linked to apoptosis independently of its canonical role as a TLR3 trafficking chaperone. Harris and Coyne showed that wild-type UNC93B1 can induce apoptotic cell death, whereas the H412R mutant, which cannot bind TLR3 or exit the ER, fails to do so. Moreover, UNC93B1 is cleaved by caspases during TRIF-mediated signaling, placing this chaperone within a proteolytic network connecting innate immune sensing and apoptotic execution. These observations suggest that UNC93B1-dependent trafficking competence may be coupled to the spatial assembly or activation of pro-death signaling platforms [87]. However, UNC93B1-dependent spatial regulation is not necessarily antitumoral in all contexts. In metastatic intestinal epithelial cells, UNC93B1 and endolysosomal acidification are required for poly(I:C)-induced CXCL10 production, yet TLR3 is also detected at the plasma membrane and can respond to extracellular dsRNA without productive IFN induction. This indicates that altered UNC93B1-dependent routing may support non-canonical TLR3 topologies in malignant cells, allowing inflammatory signaling and invasiveness rather than apoptosis [18]. Therefore, UNC93B1 should be considered a spatial gatekeeper of TLR3 signaling competence. By controlling receptor stability, trafficking, and access to endosomal or non-endosomal signaling compartments, UNC93B1 may determine whether TLR3 activation is routed toward IFN-β-associated apoptosis or toward inflammatory and tumor-adaptive programs. This positions the TLR3-UNC93B1 axis as a key regulatory node within the proposed molecular switch model.

**Figure 6:**
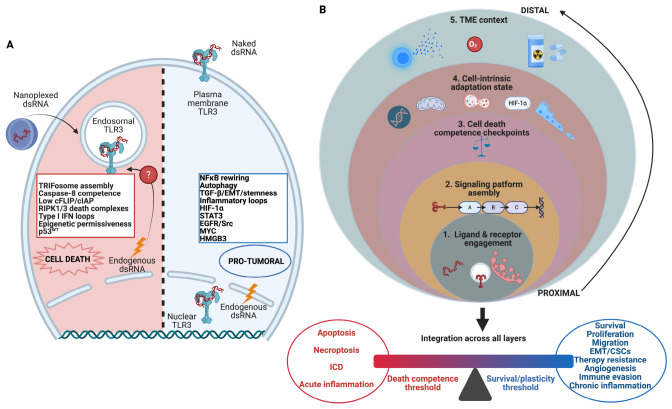
**Context-dependent determinants governing TLR3 signaling output in epithelial cancer cells.** (**A**) The figure summarizes how distinct epithelial cancer contexts may bias TLR3 signaling toward pro-death or tumor-supportive outputs. TLR3 signaling output is shaped by ligand origin, receptor localization, signaling platform composition, and intrinsic cancer cell states. Endosomal TLR3 signaling is preferentially associated with regulated cell death competence, whereas plasma membrane or nuclear-associated TLR3 signaling is linked to adaptive and tumor-supportive programs. Collectively, these determinants position TLR3 as a context-dependent molecular switch integrating innate immune sensing with cancer cell fate decisions. (**B**) Layered organization of the major determinants that bias TLR3 signaling output. These determinants include ligand source and receptor localization (1), signaling platform assembly (2), cell-death competence checkpoints (3), cell-intrinsic adaptation states (metabolism, autophagy, HIF signaling, EMT/stemness, oncogenic rewiring) (4), and TME context (5). Together, these layers establish dynamic signaling thresholds that determine whether TLR3 activation is interpreted as a death-inducing/immunogenic signal or redirected toward adaptive and tumor-promoting programs. Created in BioRender. Tittarelli, A. (2026) https://BioRender.com/8359roq.

Collectively, the available evidence suggests that the subcellular localization of TLR3 may critically influence signaling output in cancer cells, with endosomal TLR3 being preferentially associated with IFN-driven and pro-death responses, whereas plasma membrane or nuclear-associated TLR3 signaling appears more frequently linked to oncogenic, inflammatory, and tumor-adaptive programs (Figure 6).

### 5.2. Ligand Routing and Delivery-Dependent TLR3 Signaling

The biological outcome of TLR3 signaling is strongly influenced by ligand context, including its origin, intracellular routing, and delivery strategy. While cytosolic dsRNA sensors such as MDA5 and retinoic acid-inducible gene-I (RIG-I) predominantly induce type I IFN responses, engagement of endosomal TLR3 is more closely associated with apoptotic signaling [88]. Importantly, these pathways can be activated simultaneously but operate in parallel, with their relative contribution determined by ligand localization. Indeed, delivery strategies play a central role in shaping these outcomes. Naked poly(I:C) shows limited intracellular uptake and weak proapoptotic activity, whereas transfected or nanocarrier-delivered poly(I:C) enhances endosomal accumulation and TLR3 engagement. In prostate cancer models, lipofection of poly(I:C) promotes TLR3/Src-dependent apoptosis while concurrently activating cytosolic IFN pathways [88]. In this context, nanotechnology-based platforms have been explored to modulate TLR3 signaling by controlling ligand stability, uptake, and intracellular trafficking. Liposome–silica and poly(lactic-co-glycolic acid) nanoparticle (NP)-based delivery systems enhance cytotoxic and immunostimulatory activity while reducing required doses of poly(I:C). Indeed, intratumoral delivery of nanoplexed poly(I:C) induces localized tumor cell death and promotes T cell infiltration, demonstrating how spatial confinement can reshape the TME [89,90]. Similarly, combinatorial NP systems delivering TLR3 and TLR7 agonists can convert poorly immunogenic tumors into inflamed microenvironments by enhancing cytokine production and immune cell recruitment [91].

Importantly, recent evidence indicates that endogenous nucleic acid species generated by genome instability can also function as TLR3 ligands. Crossley et al. identified cytoplasmic RNA-DNA hybrids as immunogenic products derived from aberrant R-loop processing and accumulated in the cytoplasm in an endonuclease-dependent manner, indicating that these species arise through active nucleolytic processing rather than passive nuclear leakage [6]. Notably, these hybrids originated from a restricted subset of stable nuclear R-loops characterized by long half-life, partial RNase H resistance, overlapping sense-antisense hybrid formation, and specific nucleotide-skew signatures, suggesting that endogenous ligand biogenesis itself is selective and regulated. Mechanistically, accumulation of cytoplasmic RNA-DNA hybrids triggered IRF3 phosphorylation, IFN-β induction, ISG expression, and apoptosis. Importantly, cGAS and TLR3 emerged as the dominant sensors mediating this response, whereas RIG-I and MDA5 had only modest contributions. These observations establish that endogenous RNA-DNA hybrids generated during genomic instability can engage TLR3-dependent apoptotic signaling. A particularly relevant aspect of this mechanism is its spatial organization. Although TLR3 is predominantly localized within endosomal compartments, the RNA-DNA hybrids identified by Crossley et al. initially accumulate in the cytoplasm. While the precise trafficking mechanisms remain unresolved, the authors observed partial localization of cytoplasmic hybrids within endolysosomal compartments enriched for TLR3, suggesting that intracellular routing or vesicular transport may facilitate delivery of endogenous nucleic acid ligands to endosomal TLR3 signaling platforms [6]. Thus, productive TLR3 activation by endogenous ligands may require multiple sequential steps, including hybrid biogenesis, nuclear export, cytoplasmic stabilization, and access to TLR3-containing endolysosomal compartments.

Collectively, these findings support a model in which ligand origin, intracellular trafficking, and compartmental accessibility jointly determine TLR3 functional output (Figure 6). In this framework, TLR3 signaling is not simply dictated by ligand presence, but by the spatial and biochemical itinerary followed by nucleic acid ligands within the cell. Thus, endogenous ligand biogenesis and ligand routing emerge as central regulatory layers of the proposed TLR3 molecular switch in cancer cells.

### 5.3. Cancer Cell-Intrinsic States Reinterpret TLR3 Signaling Output

Beyond ligand accessibility and receptor localization, the biological consequences of TLR3 activation appear to be strongly influenced by the intrinsic molecular state of individual cancer types and cellular subpopulations. Increasing evidence indicates that TLR3 signaling does not intrinsically encode either apoptotic or tumor-promoting outputs. Instead, downstream responses emerge from the interaction between TLR3 signaling modules and pre-existing cellular programs that either favor regulated cell death or redirect signaling toward adaptive and protumoral states.

Several intracellular conditions appear to favor TLR3-mediated apoptosis and antitumor responses. The molecular composition of TLR3-associated signaling complexes plays a central role in this process. Efficient caspase-8 activation, RIPK1-dependent pro-death complex formation, low cFLIP expression, and reduced cIAPs-mediated inhibitory signaling collectively facilitate apoptotic signaling downstream of TRIF [32]. Higher-order TRIF assemblies may function as structural platforms integrating inflammatory and death-associated signaling modules, thereby enabling the transition from NFκB signaling to apoptosis [32]. In apoptosis-permissive tumor contexts, tumor suppressor pathways such as wild-type p53 may lower the threshold required for TLR3-dependent cell death [92]. Epigenetic permissiveness also contributes to productive TLR3 responses. In neuroblastoma, mesenchymal-like cells exhibit robust TLR3 responsiveness, whereas adrenergic cells remain largely unresponsive despite intact signaling machinery. Importantly, epigenetic reprogramming restores TLR3 responsiveness, demonstrating that chromatin accessibility and lineage-associated transcriptional states critically regulate TLR3 signaling competence [22]. Together with efficient endosomal ligand routing and endogenous dsRNA cytoplasmic accumulation, these cellular states appear to bias TLR3 signaling toward IRF3 activation, IFN production, and apoptotic execution.

Conversely, multiple oncogenic and adaptive programs can reinterpret TLR3 activation toward tumor-supportive outputs. In pancreatic cancer, MYC suppresses TLR3-dependent immune surveillance by limiting endogenous dsRNA trafficking and TLR3 loading, thereby promoting immune evasion [24]. In ESCC, HMGB3 interacts with TLR3 and promotes NFκB-dependent activation of TGF-β signaling, shifting signaling toward epithelial plasticity and metastasis [77]. Likewise, scaffold proteins such as β-arrestin 2 modulate TRAF6 ubiquitination and redirect TLR3 signaling toward NFκB activation and autophagy-associated survival pathways in lung cancer cells [72]. In metabolically adapted tumor states, these pathways may further reinforce tumor-promoting TLR3 outputs. Microbiota-derived SCFAs can either suppress or enhance TLR3 signaling depending on context. Activation of free fatty acid receptor 2 (FFAR2) inhibits TLR3-driven NFκB signaling in lung cancer cells, whereas in prostate cancer, SCFAs promote TLR3 expression and autophagy-dependent inflammatory signaling associated with tumor progression [67,73]. As mentioned in previous sections, additional adaptive pathways involving HIF-1α, STAT3, autophagy, EMT-associated plasticity, and inflammatory cytokine loops further amplify survival, migration, stemness, and resistance phenotypes downstream of TLR3 activation.

Collectively, our current knowledge supports a model in which intrinsic cancer cell states act as regulatory filters that bias TLR3 signaling toward either pro-death or tumor-supportive outputs. Thus, the final biological consequence of TLR3 activation is not dictated by receptor engagement alone, but rather by the balance between intracellular programs permissive for apoptotic execution and adaptive pathways that redirect signaling toward survival and tumor progression (Figure 6). This framework further supports the concept of TLR3 as a context-dependent molecular switch operating through the integration of ligand routing, spatial organization, and intrinsic cellular state.

## 6. Future Directions

Although the studies discussed above support a model in which TLR3 functions as a context-dependent molecular switch in cancer cells, several key questions remain unresolved. First, the relative hierarchy among the determinants that shape TLR3 output remains poorly defined. Spatial localization, ligand routing, signaling complex composition, and tumor-intrinsic states clearly influence TLR3 responses, but whether these factors act independently, sequentially, or hierarchically has not been systematically established. Future studies should therefore move beyond endpoint-based analyses and incorporate time-resolved approaches capable of tracking TLR3 localization, scaffold assembly, transcriptional activation, and cell fate decisions within the same experimental system.

Second, the extent to which the findings obtained with synthetic dsRNA ligands, particularly poly(I:C), reflect endogenous TLR3 activation in tumors remains incompletely understood. Endogenous dsRNA-like species derived from therapy-induced damage, epigenetic derepression, repetitive elements, extracellular vesicles, or R-loop dysregulation may differ in abundance, localization, persistence, and receptor accessibility. Defining how these endogenous ligands are generated, trafficked, and presented to TLR3 will be essential to understand whether TLR3 activation in tumors primarily reflects antiviral mimicry, sterile damage sensing, or oncogene-regulated RNA surveillance.

Third, robust biomarkers are needed to predict whether TLR3 activation will favor tumor cell death or tumor-supportive programs. Candidate determinants include TLR3 subcellular localization, TRIFosome composition, RIPK1/RIPK3/caspase-8 competence, cFLIP and cIAPs abundance, NFκB-IRF3 balance, metabolic state, and oncogenic regulators such as MYC, HIF-1α, STAT3, and EGFR. However, these variables have rarely been evaluated together. Integrative multi-omics, spatial proteomics, phosphoproteomics, and single-cell approaches applied to patient-derived models could help define predictive signatures of TLR3 responsiveness.

Therapeutic exploitation of TLR3 will require strategies that selectively bias signaling toward antitumor outputs while avoiding tumor-promoting reprogramming. This may involve optimized ligand delivery, tumor-restricted activation, combination with inhibitors of survival checkpoints, or patient selection based on molecular vulnerabilities. Importantly, future studies should distinguish cancer cell-intrinsic TLR3 signaling from TLR3 activation in immune and stromal compartments, as these may produce divergent and even opposing effects within the same tumor ecosystem.

Importantly, several methodological limitations should be considered when interpreting the current literature on TLR3 signaling in cancer cells. A major challenge is that many studies rely on poly(I:C)-based stimulation systems, which are frequently interpreted as selective models of TLR3 activation despite the ability of poly(I:C) to engage multiple dsRNA sensing pathways, including MDA5 and RIG-I. The relative contribution of these sensors may vary substantially depending on RNA length, formulation, transfection methods, NP encapsulation, endosomal delivery efficiency, and cytosolic access. Consequently, some biological effects attributed to TLR3 may instead reflect integrated responses arising from multiple dsRNA sensing systems. In addition, substantial heterogeneity exists among tumor models regarding TLR3 expression, RIPK3 competence, IFN signaling integrity, oncogenic background, metabolic state, and inflammatory adaptation. These differences complicate direct comparisons between studies and may contribute to the apparently contradictory outcomes associated with TLR3 activation across epithelial cancers. Importantly, many mechanistic observations derive from in vitro systems that incompletely recapitulate the complexity of tumor-immune interactions occurring in vivo, particularly regarding ICD, cytokine-mediated signaling amplification, and stromal or immune cell-dependent effects.

Together, these limitations emphasize that TLR3 signaling outputs should be interpreted within the specific experimental context in which they were observed and highlight the need for comparative, multi-model, and time-resolved approaches capable of distinguishing tumor-intrinsic TLR3 functions from broader dsRNA sensing responses.

## 7. Translational Challenges and Therapeutic Perspectives of TLR3 Targeting in Epithelial Cancers

The capacity of TLR3 activation to simultaneously induce cancer cell death, enhance antigen presentation, promote type I IFN production, and stimulate antitumor immunity has positioned TLR3 agonists as attractive candidates for cancer therapy and, particularly, immunotherapy. Over the past decades, multiple synthetic dsRNA agonists, including poly(I:C), poly-ICLC (Hiltonol), poly(A:U), Rintatolimod (Ampligen), and BO-112, have demonstrated promising antitumor activity in preclinical models and have progressively entered clinical evaluation as monotherapies, vaccine adjuvants, intratumoral immunotherapies, or combinatorial approaches with chemotherapy, radiotherapy, and immune checkpoint blockade [4,10,11,93,94].

Several trends emerge from the current clinical trial landscape for epithelial cancers summarized in Table 3. First, most ongoing therapeutic strategies do not employ TLR3 agonists as standalone agents, but rather as components of combinatorial therapy regimens. TLR3 agonists are increasingly being combined with immune checkpoint blockade, chemotherapy, cancer vaccines, or cytokine modulation approaches, reflecting the growing recognition that TLR3 activation primarily functions as an immune-amplifying and immunomodulatory platform. Second, many current protocols utilize intratumoral, locoregional, or NP-assisted delivery strategies instead of conventional systemic administration. This shift likely reflects attempts to maximize local cancer cell death and immunostimulatory effects while minimizing systemic inflammatory toxicity and off-target responses associated with circulating dsRNA agonists.

Nevertheless, the clinical development of TLR3 agonists illustrates a persistent gap between strong biological rationale and variable therapeutic efficacy. Earlier clinical experience with dsRNA agonists already suggested that antitumor activity is highly dependent on formulation, disease context, and treatment setting. For example, poly(A:U) showed encouraging long-term outcomes as adjuvant therapy in operable breast cancer, with improved 5-year overall survival and relapse-free survival compared with standard treatment and improved long-term survival when combined with 5-fluorouracil and doxorubicin in locally advanced gastric cancer [95,96]. In contrast, other regimens have shown limited efficacy, such as cytokine-modulating therapy including rintatolimod in colorectal cancer metastatic to the liver, which yielded an objective response rate of 0% and a short median progression-free survival [97]. These mixed outcomes indicate that TLR3 agonists should not be considered broadly effective immunostimulants across epithelial cancers, but rather context-sensitive agents whose activity depends on the tumor immune landscape, delivery route, agonist formulation, and the presence of complementary therapies capable of converting innate immune activation into durable adaptive antitumor responses. This interpretation is further supported by more recent clinical and translational studies. Intratumoral poly-ICLC combined with tremelimumab and systemic durvalumab in recurrent breast cancer induced clinical responsiveness together with increased infiltration or activation of CD8^+^ T cells, CD20^+^ B cells, mature DC, macrophages, and CD56^+^ NK cells, suggesting that localized TLR3 activation can remodel the TME when embedded within checkpoint blockade strategies [98]. Similarly, BO-112, a nanoplexed poly(I:C) formulation, was developed to enhance intratumoral innate immune activation [99]. In anti-PD-1-refractory advanced melanoma, intratumoral BO-112 combined with pembrolizumab produced an objective response rate of 27%, stable disease in 37.8% of patients, and two pathological complete responses, although treatment-related adverse events were frequent and grade 3–5 events occurred in 36% of patients [100,101]. These data illustrate both the promise and the risks of TLR3-directed therapy: local delivery can generate clinically meaningful immune activation, but toxicity and patient selection remain key barriers.

An additional challenge relates to the pharmacological and delivery-associated limitations of synthetic dsRNA agonists. Systemic administration of poly(I:C)-based compounds is frequently associated with excessive inflammatory responses, cytokine-associated toxicities, poor tumor selectivity, rapid degradation, and suboptimal pharmacokinetic properties, all of which may limit therapeutic window and clinical applicability. Moreover, because dsRNA molecules can activate additional cytosolic RNA sensors, including MDA5 and RIG-I, the biological effects of poly(I:C)-based therapies may extend beyond TLR3 itself, further increasing signaling complexity and systemic inflammatory toxicity [4,10]. Importantly, emerging evidence suggests that delivery strategy critically influences TLR3 therapeutic output. Intratumoral administration and nanoplexed dsRNA formulations appear particularly promising because they may enhance local immune activation while minimizing systemic inflammatory toxicity. In this context, BO-112, a nanoplexed formulation of poly(I:C), has shown encouraging activity in preclinical and early clinical settings, particularly in combination with PD-1 blockade in anti-PD-1-resistant melanoma [100]. Similarly, chemically engineered agonists such as TL-532 were developed to improve pharmacological properties and reduce off-target effects associated with structurally homogeneous dsRNA compounds [102].

## 8. Conclusions: TLR3 as a Molecular Switch

The evidence discussed in this review supports an integrative model in which TLR3 may function as a context-dependent molecular switch in epithelial cancers. However, this model should be interpreted as a conceptual synthesis rather than as a universally established mechanism, because many of the conclusions derive from heterogeneous studies performed in different tumor models, ligand systems, and experimental settings. Within defined experimental contexts, several mechanisms are well supported, including TRIF-dependent IRF3/NFκB activation, assembly of RIPK1/FADD/caspase-8-associated death complexes, RIPK3/MLKL-dependent necroptotic competence, and regulation by inhibitory checkpoints such as cFLIP and cIAPs. By contrast, the broader idea that these determinants operate as a unified hierarchical switch across epithelial cancers remains a working framework that requires direct comparative validation.

At the molecular level, TLR3 signaling is organized around a modular architecture in which distinct functional outputs emerge from the selective engagement of interconnected signaling branches. The TRIF-dependent platform serves as a central scaffold that can recruit and assemble alternative signaling complexes, including RIPK1–FADD–caspase-8 modules that drive apoptosis, RIPK1-RIPK3-MLKL complexes that enable necroptosis, and TRAF-dependent assemblies that activate NFκB and IRF3. The relative dominance of these modules is governed by intracellular checkpoints such as cFLIP, cIAPs, and RIPK1, which define the threshold for cell death activation versus survival signaling. Importantly, these signaling branches are not mutually exclusive but coexist within a highly interconnected network in which transcriptional, metabolic, and inflammatory pathways dynamically interact. NFκB-driven inflammatory programs can simultaneously promote survival and sensitize cells to apoptosis depending on the balance of downstream targets, while IRF3-mediated IFN responses can cooperate with or counteract inflammatory signaling. In parallel, metabolic regulators such as HIF-1α and MYC further reprogram TLR3 outputs by linking innate immune sensing to cellular adaptation and plasticity.

Spatial determinants critically contribute to this switch-like behavior. The subcellular localization of TLR3 (whether confined to endosomes, redistributed to the plasma membrane, or translocated to the nucleus) defines receptor accessibility, adaptor usage, and downstream signaling topology. Likewise, ligand context and delivery mechanisms determine which dsRNA sensors are engaged and how signals are routed intracellularly, thereby biasing the balance between apoptotic, immunostimulatory, or tumor-promoting responses.

At the systems level, TLR3 signaling can be conceptualized as a multi-input decision-making network that governs cell fate transitions. Under conditions where pro-death signaling thresholds are exceeded, TLR3 activation drives RCD. Conversely, when survival pathways, metabolic adaptation, or oncogenic rewiring dominate, TLR3 signaling is redirected toward tumor-promoting programs, including proliferation, epithelial plasticity, stemness, and pro-inflammatory microenvironmental remodeling. These alternative outcomes are stabilized by feedback loops that reinforce specific cellular states, enabling dynamic but structured transitions between tumor-suppressive and tumor-promoting phenotypes.

In this framework, the apparent duality of TLR3 signaling is not paradoxical but reflects its inherent capacity to function as a programmable molecular switch. Understanding the regulatory logic governing this switch is essential for the rational design of therapeutic strategies aimed at selectively biasing TLR3 signaling toward antitumor outcomes. Targeting key determinants such as signaling scaffold composition, ligand delivery, metabolic state, or microenvironmental context may allow the reprogramming of TLR3 activity from a tumor-supportive pathway into a potent driver of cancer cell death and immune activation.

## Figures and Tables

**Table 1:** **Operational classification of canonical and non-canonical TLR3 signaling modules in epithelial cancer cells.**

Pathway Module	Classification	TRIF Dependence	Transcription Dependence	Main Molecular Components	Dominant Output
TRIF-IRF3	Canonical	Yes	Yes	TRIF, TRAF3, TBK1, IRF3	Type I IFN, ISGs
TRIF-NFκB				TRIF, TRAF6, TAK1, IKK, NFκB	Inflammatory cytokines, chemokines
Trifosome-NFκB			Mostly yes	TRIF oligomers, RIPK1, RIPK3, ZBP1	Signal amplification, cell-fate modulation
TRIF-caspase-8	Non-canonical death-associated		Partially/no	TRIF, RIPK1, FADD, caspase-8, cIAPs, cFLIP	Apoptosis
TRIF-MLKL				TRIF, RIPK3, MLKL, RIPK1	Necroptosis
TLR3-Src	Non-canonical kinase-associated	No/low	No	Src, lipid rafts,	Cell migration and proliferation changes
TLR3-EGFR-Src	Non-canonical regulatory/kinase-associated	Partial/context-dependent	Both	EGFR, Src, TRIF	TLR3 phosphorylation, cell plasticity, transcriptional licensing
Autophagy-NFκB	Context-dependent non-canonical modifier	Indirect/variable	Yes	Autophagy machinery, TRAF6, NFκB	Sustained inflammatory/protumoral signaling

cFLIP: cellular FLICE-like inhibitory protein; cIAPs: cellular inhibitor of apoptosis proteins; EGFR: epidermal growth factor receptor; FADD: fas-associated protein with dead domain; IKK: IκB kinase; IRF3: interferon (IFN) regulatory factor 3; ISGs: IFN-stimulated genes; MLKL: mixed linage kinase domain-like protein; NFκB: nuclear factor kappa B; RIPK: receptor-interacting serine/threonine kinase; TAK1: transforming growth factor-β-activated kinase 1; TBK1: TANK-binding kinase 1; TRAF: tumor necrosis factor receptor-associated factor; TRIF: TIR-domain-containing adapter-inducing IFN-β; ZBP1: Z-DNA-binding protein 1.

**Table 2:** **Epithelial cancer-type-specific outputs of TLR3 signaling.**

Tumor Context	Predominant TLR3-Associated Output	Main Molecular Determinants
Breast cancer	Apoptosis or stemness/progression	IFN/TRAIL loops, NFκB, Wnt/β-catenin, HIF-1α
Prostate cancer	Apoptosis or angiogenesis/invasion	PKC/MAPK, HIF-1α, SCAF/autophagy
Colorectal cancer	Apoptosis/necroptosis or chemoresistance	UNC93B1, TAK1, RIPK1/RIPK3, NFκB
Head and neck squamous cell carcinoma	Apoptosis or chemoresistance	NFκB, DAMP, IFN/CCL5
Lung cancer	Apoptosis or invasion/migration	cFLIP/cIAPs, IL-6/JAK2/STAT3, FFAR2, Scr
Pancreatic cancer	Immune surveillance or tumor adaptation	MYC/MIZ1, endogenous dsRNA
Ovarian cancer	IFN/apoptosis or EMT/invasion	Src/Syk/PI3K, endogenous dsRNA
Hepatocellular carcinoma	Apoptosis/ferroptosis or immune escape	TLR3 expression, autophagy-dependent TRIF degradation

**Table 3:** **Clinical trials currently testing TLR3 agonists in epithelial cancers.**

Agonist	Indication	Phase	Status	Co-Therapy	NCT Number
Rintatolimod	Metastatic pancreatic cancer	Ib	Recruiting	Anti-PD-L1	NCT05927142
	Ovarian cancer recurrent	II	Not recruiting	Cisplatin, anti-PD-1	NCT03734692
	Prostate cancer (stage IIA)	II	Not recruiting	Aspirin, IFN-α 2b	NCT03899987
BO-112	Basal cell carcinoma	IIb	Not recruiting	Single agent	NCT06422936
Poly-ICLC	Prostate cancer	I	Not recruiting	Multi-peptide vaccine	NCT05010200
	Prostate cancer	II	Recruiting	Single agent (im/it)	NCT06343077
	Colorectal and pancreatic cancer (stage IV)	Ib	Recruiting	Peptide vaccine, anti-PD-1, anti-CTLA-4	NCT06411691
	Pancreatic cancer	I	Recruiting	Peptide vaccine	NCT05013216
	Lung carcinoma	I	Not recruiting	Peptide vaccine	NCT03300817
	Gastroesophageal adenocarcinoma and muscle-invasive bladder carcinoma	I	Recruiting	Personalized peptide vaccine	NCT06529822
	Breast cancer (stage IV) and others	I	Not recruiting	Flt3L, CD40 agonist, anti-PD-1, anti-IL-6	NCT04616248
	Biliary tract cancer	I	Recruiting	Peptide vaccine, anti-PD-L1, anti-CTLA-4	NCT06564623
	Metastatic melanoma, breast (HER2^neg^), and non-small-cell lung cancer	I	Recruiting	Personalized neoAg vaccine	NCT05098210
	Solid tumors	I/II	Recruiting	Personalized neoAg vaccine, anti-PD-1	NCT07002203
	Colorectal and pancreatic cancer	I	Not recruiting	Peptide vaccine, anti-PD-1, anti-CTLA-4	NCT04117087
	Colorectal cancer	II	Not recruiting	Peptide vaccine	NCT02134925
	Breast cancer (TNBC) (stages II/III)	I	Not recruiting	Anti-PD-L1, vaccine	NCT02826434
	Ductal carcinoma in situ	I	Recruiting	Peptide vaccine, aromatase inhibitor, selective estrogen receptor modulator	NCT06218303
	Fibrolamellar hepatocellular carcinoma	I	Recruiting	Peptide vaccine, anti-PD-1, anti-CTLA-4	NCT04248569
	Pancreatic cancer	I/II	Recruiting	Whole tumor cell vaccine, peptide vaccine, anti-PD-1, anti-CD137	NCT06782932
	Kidney cancer	I	Not recruiting	Personalized NeoAg vaccine, anti-CTLA-4	NCT02950766
	Non-small-cell lung cancer	I	Recruiting	Peptide vaccine	NCT05254184
	Ovarian cancer	I	Not recruiting	Personalized NeoAg vaccine, anti-PD-1	NCT04024878
	Ovarian cancer	II/III	Recruiting	Personalized NeoAg vaccine	NCT06341907
	Non-small-cell lung cancer	I/II	Recruiting	Peptide vaccine	NCT01720836
	Breast cancer (stage IV)	II	Recruiting	Carboplatin, gemcitabine, paclitaxel, personalized peptide vaccine, anti-PD-L1, anti-Trop-2, anti-CTLA-4	NCT03606967

BO-112: poly(I:C) nanoplexed with polyethylenimine; CTLA-4: cytotoxic T-lymphocyte-associated protein-4; Flt3L: Fms-like tyrosine kinase 3 ligand; im: intramuscular; it: intratumoral; NeoAg: neoantigen; PD-1: programmed death protein-1; PD-L1: programmed death-ligand 1; Poly-ICLC (Hiltonol): synthetic complex of carboxymethylcellulose, poly(I:C), and poly-L-lysine; Rintatolimod (Ampligen): analog to poly(I:C) (PolyI: PolyC_12_U); TNBC: triple negative breast cancer; Trop-2: trophoblast cell surface antigen-2.

## Data Availability

No new data were created or analyzed in this study. Data sharing is not applicable to this article.

## Abbreviations

The following abbreviations are used in this manuscript:

AMPK	AMP-activated protein kinase
APC	Antigen-presenting cell
cAMP	Cyclic adenosine monophosphate
CCL	C-C motif chemokine ligand
cFLIP	Cellular FLICE-like inhibitory protein
cIAPs	Cellular inhibitor of apoptosis proteins
CSC	Cancer stem cell
CXCL	C-X-C motif chemokine ligand
DAMPs	Danger-associated molecular patterns
DC	Dendritic cell
dsRNA	Double-stranded RNA
EGFR	Epidermal growth factor receptor
EMT	Epithelial-to-mesenchymal transition
ERK	Extracellular signal-regulated kinase
ESCC	Esophageal squamous cell carcinoma
FADD	Fas-associated protein with death domain
FFAR2	Free fatty acid receptor 2
HCC	Hepatocellular carcinoma
HIF-1α	Hypoxia-inducible factor 1-alpha
HNSCC	Head and neck squamous cell carcinoma
ICD	Immunogenic cell death
IFN	Interferon
IKK	IκB kinase
IL	Interleukin
IRAK	Interleukin-1 receptor-associated kinase
IRF3	Interferon regulatory factor 3
ISG	Interferon-stimulated gene
JAK	Janus kinase
JNK	c-Jun N-terminal kinase
MAPK	Mitogen-activated protein kinase
MDA5	Melanoma differentiation-associated protein 5
MLKL	Mixed lineage kinase domain-like protein
MYC	MYC proto-oncogene
NFκB	Nuclear factor kappa B
NK	Natural killer
NSCLC	Non-small-cell lung cancer
PARP	Poly(ADP-ribose) polymerase
PI3K	Phosphoinositide 3-kinase
PKC	Protein kinase C
poly(I:C)	Polyinosinic:polycytidylic acid
PRR	Pattern recognition receptor
RCD	Regulated cell death
RIG-I	Retinoic acid-inducible gene I
RIPK	Receptor-interacting serine/threonine kinase
RHIM	RIP homotypic interaction motif
ROS	Reactive oxygen species
SCFA	Short-chain fatty acid
Src	Proto-oncogene tyrosine-protein kinase Src
STAT3	Signal transducer and activator of transcription 3
TAK1	Transforming growth factor-β-activated kinase 1
TBK1	TANK-binding kinase 1
TGF-β	Transforming growth factor beta
TIR	Toll/interleukin-1 receptor
TLR3	Toll-like receptor 3
TME	Tumor microenvironment
TNF	Tumor necrosis factor
TRAF	TNF receptor-associated factor
TRAIL	TNF-related apoptosis-inducing ligand
TRIF	TIR-domain-containing adapter-inducing interferon-β
VEGF	Vascular endothelial growth factor
Wnt	Wingless-related integration site
ZBP1	Z-DNA-binding protein 1

## References

[1] Seelige R., Searles S., Bui J.D. (2018). Innate sensing of cancer’s non-immunologic hallmarks. Curr. Opin. Immunol..

[2] Man S.M., Jenkins B.J. (2022). Context-dependent functions of pattern recognition receptors in cancer. Nat. Rev. Cancer.

[3] Pradere J.P., Dapito D.H., Schwabe R.F. (2014). The Yin and Yang of Toll-like receptors in cancer. Oncogene.

[4] Hsieh M.L., Nishizaki D., Adashek J.J., Kato S., Kurzrock R. (2025). Toll-like receptor 3: A double-edged sword. Biomark. Res..

[5] Alexopoulou L., Holt A.C., Medzhitov R., Flavell R.A. (2001). Recognition of double-stranded RNA and activation of NF-kappaB by Toll-like receptor 3. Nature.

[6] Crossley M.P., Song C., Bocek M.J., Choi J.H., Kousouros J.N., Sathirachinda A., Lin C., Brickner J.R., Bai G., Lans H. (2023). R-loop-derived cytoplasmic RNA-DNA hybrids activate an immune response. Nature.

[7] Luan X., Wang L., Song G., Zhou W. (2024). Innate immune responses to RNA: Sensing and signaling. Front. Immunol..

[8] Kaiser W.J., Offermann M.K. (2005). Apoptosis induced by the toll-like receptor adaptor TRIF is dependent on its receptor interacting protein homotypic interaction motif. J. Immunol..

[9] Imre G., Larisch S., Rajalingam K. (2011). Ripoptosome: A novel IAP-regulated cell death-signalling platform. J. Mol. Cell Biol..

[10] Le Naour J., Galluzzi L., Zitvogel L., Kroemer G., Vacchelli E. (2020). Trial watch: TLR3 agonists in cancer therapy. Oncoimmunology.

[11] Butkowsky C., Aldor N., Poynter S.J. (2023). Toll-like receptor 3 ligands for breast cancer therapies (Review). Mol. Clin. Oncol..

[12] Paone A., Starace D., Galli R., Padula F., De Cesaris P., Filippini A., Ziparo E., Riccioli A. (2008). Toll-like receptor 3 triggers apoptosis of human prostate cancer cells through a PKC-alpha-dependent mechanism. Carcinogenesis.

[13] Salaun B., Coste I., Rissoan M.C., Lebecque S.J., Renno T. (2006). TLR3 can directly trigger apoptosis in human cancer cells. J. Immunol..

[14] Estornes Y., Toscano F., Virard F., Jacquemin G., Pierrot A., Vanbervliet B., Bonnin M., Lalaoui N., Mercier-Gouy P., Pachéco Y. (2012). dsRNA induces apoptosis through an atypical death complex associating TLR3 to caspase-8. Cell Death Differ..

[15] Sistigu A., Yamazaki T., Vacchelli E., Chaba K., Enot D.P., Adam J., Vitale I., Goubar A., Baracco E.E., Remédios C. (2014). Cancer cell-autonomous contribution of type I interferon signaling to the efficacy of chemotherapy. Nat. Med..

[16] Conrad M., Strasser A., Jost P.J., Yuan J., Shao F., Vandenabeele P., Wahida A. (2026). Cell death in cancer. Cell.

[17] Jia D., Yang W., Li L., Liu H., Tan Y., Ooi S., Chi L., Filion L.G., Figeys D., Wang L. (2015). β-Catenin and NF-κB co-activation triggered by TLR3 stimulation facilitates stem cell-like phenotypes in breast cancer. Cell Death Differ..

[18] Bugge M., Bergstrom B., Eide O.K., Solli H., Kjønstad I.F., Stenvik J., Espevik T., Nilsen N.J. (2017). Surface Toll-like receptor 3 expression in metastatic intestinal epithelial cells induces inflammatory cytokine production and promotes invasiveness. J. Biol. Chem..

[19] Feoktistova M., Geserick P., Kellert B., Dimitrova D.P., Langlais C., Hupe M., Cain K., MacFarlane M., Häcker G., Leverkus M. (2011). cIAPs block Ripoptosome formation, a RIP1/caspase-8 containing intracellular cell death complex differentially regulated by cFLIP isoforms. Mol. Cell.

[20] Alkurdi L., Virard F., Vanbervliet B., Weber K., Toscano F., Bonnin M., Le Stang N., Lantuejoul S., Micheau O., Renno T. (2018). Release of c-FLIP brake selectively sensitizes human cancer cells to TLR3-mediated apoptosis. Cell Death Dis..

[21] Tummers B., Green D.R. (2022). Mechanisms of TNF-independent RIPK3-mediated cell death. Biochem. J..

[22] Wolpaw A.J., Grossmann L.D., Dessau J.L., Dong M.M., Aaron B.J., Brafford P.A., Volgina D., Pascual-Pasto G., Rodriguez-Garcia A., Uzun Y. (2022). Epigenetic state determines inflammatory sensing in neuroblastoma. Proc. Natl. Acad. Sci. USA.

[23] Chen Y.G., Hur S. (2022). Cellular origins of dsRNA, their recognition and consequences. Nat. Rev. Mol. Cell Biol..

[24] Krenz B., Gebhardt-Wolf A., Ade C.P., Gaballa A., Roehrig F., Vendelova E., Baluapuri A., Eilers U., Gallant P., D’Artista L. (2021). MYC- and MIZ1-Dependent Vesicular Transport of Double-Strand RNA Controls Immune Evasion in Pancreatic Ductal Adenocarcinoma. Cancer Res..

[25] Muresan X.M., Bouchal J., Culig Z., Souček K. (2020). Toll-Like Receptor 3 in Solid Cancer and Therapy Resistance. Cancers.

[26] Wang Z., Gu Y., Liu Y., Wang Z., Chen X., Wang H., Zhang W., Jin G., Cao X. (2025). Phosphorylated Toll-like receptor 3 nuclear translocation in cancer cell promotes metastasis and chemoresistance. Signal Transduct. Target. Ther..

[27] Lim C.S., Jang Y.H., Lee G.Y., Han G.M., Jeong H.J., Kim J.W., Lee J.O. (2022). TLR3 forms a highly organized cluster when bound to a poly(I:C) RNA ligand. Nat. Commun..

[28] Vercammen E., Staal J., Beyaert R. (2008). Sensing of viral infection and activation of innate immunity by toll-like receptor 3. Clin. Microbiol. Rev..

[29] Xu J., Hu Q., Zhu Y., Liu Q., Wang F., Yu Y., Wang W., Ding X. (2026). The TRIM3/TLR3 axis overrides IFN-β feedback inhibition to suppress NSCLC progression. Cell Death Dis..

[30] Popli S., Chakravarty S., Fan S., Glanz A., Aras S., Nagy L.E., Sen G.C., Chakravarti R., Chattopadhyay S. (2022). IRF3 inhibits nuclear translocation of NF-κB to prevent viral inflammation. Proc. Natl. Acad. Sci. USA.

[31] Muendlein H.I., Connolly W.M., Magri Z., Jetton D., Smirnova I., Degterev A., Balachandran S., Poltorak A. (2022). ZBP1 promotes inflammatory responses downstream of TLR3/TLR4 via timely delivery of RIPK1 to TRIF. Proc. Natl. Acad. Sci. USA.

[32] Moncrieffe M.C., Suresh P., Boyle J., Cui Y., Nawalpuri B., Verstak B., Zhang Y.P., Zhang Z., Taylor M., Egelman E.H. (2026). Toll-like receptor signaling outcome is determined by the stoichiometry of the endogenous TRIFosome. Sci. Adv..

[33] Yamashita M., Chattopadhyay S., Fensterl V., Zhang Y., Sen G.C. (2012). A TRIF-independent branch of TLR3 signaling. J. Immunol..

[34] Yamashita M., Chattopadhyay S., Fensterl V., Saikia P., Wetzel J.L., Sen G.C. (2012). Epidermal growth factor receptor is essential for Toll-like receptor 3 signaling. Sci. Signal..

[35] Salaun B., Zitvogel L., Asselin-Paturel C., Morel Y., Chemin K., Dubois C., Massacrier C., Conforti R., Chenard M.P., Sabourin J.C. (2011). TLR3 as a biomarker for the therapeutic efficacy of double-stranded RNA in breast cancer. Cancer Res..

[36] An Y., Wang X., Wu X., Chen L., Yang Y., Lin X., Wang N., Duan J., Long S., Zhao X. (2021). Oncolytic reovirus induces ovarian cancer cell apoptosis in a TLR3-dependent manner. Virus Res..

[37] Jiang Q., Wei H., Tian Z. (2008). Poly I:C enhances cycloheximide-induced apoptosis of tumor cells through TLR3 pathway. BMC Cancer.

[38] Bernardo A.R., Cosgaya J.M., Aranda A., Jiménez-Lara A.M. (2013). Synergy between RA and TLR3 promotes type I IFN-dependent apoptosis through upregulation of TRAIL pathway in breast cancer cells. Cell Death Dis..

[39] Bernardo A.R., Cosgaya J.M., Aranda A., Jiménez-Lara A.M. (2017). Pro-apoptotic signaling induced by Retinoic acid and dsRNA is under the control of Interferon Regulatory Factor-3 in breast cancer cells. Apoptosis.

[40] Zhao J., Xue Y., Pan Y., Yao A., Wang G., Li D., Wang T., Zhao S., Hou Y. (2019). Toll-like receptor 3 agonist poly I:C reinforces the potency of cytotoxic chemotherapy via the TLR3-UNC93B1-IFN-β signaling axis in paclitaxel-resistant colon cancer. J. Cell Physiol..

[41] Weiss R., Sachet M., Zinngrebe J., Aschacher T., Krainer M., Hegedus B., Walczak H., Bergmann M. (2013). IL-24 sensitizes tumor cells to TLR3-mediated apoptosis. Cell Death Differ..

[42] Kourko O., Smyth R., Cino D., Seaver K., Petes C., Eo S.Y., Basta S., Gee K. (2019). Poly(I:C)-Mediated Death of Human Prostate Cancer Cell Lines Is Induced by Interleukin-27 Treatment. J. Interf. Cytokine Res..

[43] Ma Y.F., Ren Y., Wu C.J., Zhao X.H., Xu H., Wu D.Z., Xu J., Zhang X.L., Ji Y. (2016). Interleukin (IL)-24 transforms the tumor microenvironment and induces anticancer immunity in a murine model of colon cancer. Mol. Immunol..

[44] Bréart B., Williams K., Krimm S., Wong T., Kayser B.D., Wang L., Cheng E., Cruz Tleugabulova M., Bouziat R., Lu T. (2025). IL-27 elicits a cytotoxic CD8+ T cell program to enforce tumour control. Nature.

[45] Umemura N., Zhu J., Mburu Y.K., Forero A., Hsieh P.N., Muthuswamy R., Kalinski P., Ferris R.L., Sarkar S.N. (2012). Defective NF-κB signaling in metastatic head and neck cancer cells leads to enhanced apoptosis by double-stranded RNA. Cancer Res..

[46] McCormick K.D., Ghosh A., Trivedi S., Wang L., Coyne C.B., Ferris R.L., Sarkar S.N. (2016). Innate immune signaling through differential RIPK1 expression promote tumor progression in head and neck squamous cell carcinoma. Carcinogenesis.

[47] Harashima N., Inao T., Imamura R., Okano S., Suda T., Harada M. (2012). Roles of the PI3K/Akt pathway and autophagy in TLR3 signaling-induced apoptosis and growth arrest of human prostate cancer cells. Cancer Immunol. Immunother..

[48] Wang G., Zhang M., Li Y., Zhou J., Chen L. (2017). Studying the Effect of Downregulating Autophagy-Related Gene LC3 on TLR3 Apoptotic Pathway Mediated by dsRNA in Hepatocellular Carcinoma Cells. Cancer Res. Treat..

[49] Bonnin M., Fares N., Testoni B., Estornes Y., Weber K., Vanbervliet B., Lefrançois L., Garcia A., Kfoury A., Pez F. (2019). Toll-like receptor 3 downregulation is an escape mechanism from apoptosis during hepatocarcinogenesis. J. Hepatol..

[50] Weber A., Kirejczyk Z., Besch R., Potthoff S., Leverkus M., Häcker G. (2010). Proapoptotic signalling through Toll-like receptor-3 involves TRIF-dependent activation of caspase-8 and is under the control of inhibitor of apoptosis proteins in melanoma cells. Cell Death Differ..

[51] Vanbervliet-Defrance B., Delaunay T., Daunizeau T., Kepenekian V., Glehen O., Weber K., Estornes Y., Ziverec A., Djemal L., Delphin M. (2020). Cisplatin unleashes Toll-like receptor 3-mediated apoptosis through the downregulation of c-FLIP in malignant mesothelioma. Cancer Lett..

[52] Wallach D., Kang T.B., Dillon C.P., Green D.R. (2016). Programmed necrosis in inflammation: Toward identification of the effector molecules. Science.

[53] Kaiser W.J., Sridharan H., Huang C., Mandal P., Upton J.W., Gough P.J., Sehon C.A., Marquis R.W., Bertin J., Mocarski E.S. (2013). Toll-like receptor 3-mediated necrosis via TRIF, RIP3, and MLKL. J. Biol. Chem..

[54] Dillon C.P., Weinlich R., Rodriguez D.A., Cripps J.G., Quarato G., Gurung P., Verbist K.C., Brewer T.L., Llambi F., Gong Y.N. (2014). RIPK1 blocks early postnatal lethality mediated by caspase-8 and RIPK3. Cell.

[55] Cai J., Hu D., Sakya J., Sun T., Wang D., Wang L., Mao X., Su Z. (2021). ABIN-1 is a key regulator in RIPK1-dependent apoptosis (RDA) and necroptosis, and ABIN-1 deficiency potentiates necroptosis-based cancer therapy in colorectal cancer. Cell Death Dis..

[56] Takemura R., Takaki H., Okada S., Shime H., Akazawa T., Oshiumi H., Matsumoto M., Teshima T., Seya T. (2015). PolyI:C-Induced, TLR3/RIP3-Dependent Necroptosis Backs Up Immune Effector-Mediated Tumor Elimination In Vivo. Cancer Immunol. Res..

[57] Sae-Fung A., Lomphithak T., Duangthim N., Jitkaew S. (2025). IFN-γ enhances Poly(I:C)-induced necroptosis and immunogenic cell death via TLR3 upregulation in cholangiocarcinoma cells. Eur. J. Pharm. Sci..

[58] Chiappinelli K.B., Strissel P.L., Desrichard A., Li H., Henke C., Akman B., Hein A., Rote N.S., Cope L.M., Snyder A. (2015). Inhibiting DNA Methylation Causes an Interferon Response in Cancer via dsRNA Including Endogenous Retroviruses. Cell.

[59] Mannion J., Gifford V., Bellenie B., Fernando W., Ramos Garcia L., Wilson R., John S.W., Udainiya S., Patin E.C., Tiu C. (2024). A RIPK1-specific PROTAC degrader achieves potent antitumor activity by enhancing immunogenic cell death. Immunity.

[60] Qiu L., Ji H., Wang K., Liu W., Huang Q., Pan X., Ye H., Li Z., Chen G., Xing X. (2024). TLR3 activation enhances abscopal effect of radiotherapy in HCC by promoting tumor ferroptosis. EMBO Mol. Med..

[61] Schwartz A.L., Malgor R., Dickerson E., Weeraratna A.T., Slominski A., Wortsman J., Harii N., Kohn A.D., Moon R.T., Schwartz F.L. (2009). Phenylmethimazole decreases Toll-like receptor 3 and noncanonical Wnt5a expression in pancreatic cancer and melanoma together with tumor cell growth and migration. Clin. Cancer Res..

[62] Schwartz A.L., Dickerson E., Dagia N., Malgor R., McCall K.D. (2017). TLR signaling inhibitor, phenylmethimazole, in combination with tamoxifen inhibits human breast cancer cell viability and migration. Oncotarget.

[63] Chuang H.C., Chou M.H., Chien C.Y., Chuang J.H., Liu Y.L. (2018). Triggering TLR3 pathway promotes tumor growth and cisplatin resistance in head and neck cancer cells. Oral. Oncol..

[64] Paone A., Galli R., Gabellini C., Lukashev D., Starace D., Gorlach A., De Cesaris P., Ziparo E., Del Bufalo D., Sitkovsky M.V. (2010). Toll-like receptor 3 regulates angiogenesis and apoptosis in prostate cancer cell lines through hypoxia-inducible factor 1 alpha. Neoplasia.

[65] Scatozza F., D’Amore A., Fontanella R.A., DE Cesaris P., Marampon F., Padula F., Ziparo E., Riccioli A., Filippini A. (2020). Toll-Iike Receptor-3 Activation Enhances Malignant Traits in Human Breast Cancer Cells Through Hypoxia-inducible Factor-1α. Anticancer Res..

[66] Wang X., Zhang Z., Cao H., Niu W., Li M., Xi X., Wang J. (2017). Human papillomavirus type 16 E6 oncoprotein promotes proliferation and invasion of non-small cell lung cancer cells through Toll-like receptor 3 signaling pathway. J. Med. Virol..

[67] Kim M.J., Kim J.Y., Shin J.H., Kang Y., Lee J.S., Son J., Jeong S.K., Kim D., Kim D.H., Chun E. (2023). FFAR2 antagonizes TLR2- and TLR3-induced lung cancer progression via the inhibition of AMPK-TAK1 signaling axis for the activation of NF-κB. Cell Biosci..

[68] Lomphithak T., Choksi S., Mutirangura A., Tohtong R., Tencomnao T., Usubuchi H., Unno M., Sasano H., Jitkaew S. (2020). Receptor-interacting protein kinase 1 is a key mediator in TLR3 ligand and Smac mimetic-induced cell death and suppresses TLR3 ligand-promoted invasion in cholangiocarcinoma. Cell Commun. Signal..

[69] Muresan X.M., Slabáková E., Procházková J., Drápela S., Fedr R., Pícková M., Vacek O., Víchová R., Suchánková T., Bouchal J. (2022). Toll-Like Receptor 3 Overexpression Induces Invasion of Prostate Cancer Cells, whereas Its Activation Triggers Apoptosis. Am. J. Pathol..

[70] Chen L., Feng Z., Yue H., Bazdar D., Mbonye U., Zender C., Harding C.V., Bruggeman L., Karn J., Sieg S.F. (2018). Exosomes derived from HIV-1-infected cells promote growth and progression of cancer via HIV TAR RNA. Nat. Commun..

[71] Zhan Z., Xie X., Cao H., Zhou X., Zhang X.D., Fan H., Liu Z. (2014). Autophagy facilitates TLR4- and TLR3-triggered migration and invasion of lung cancer cells through the promotion of TRAF6 ubiquitination. Autophagy.

[72] Kim J.Y., Shin J.H., Kim M.J., Kang Y., Lee J.S., Son J., Jeong S.K., Kim D., Kim D.H., Chun E. (2023). β-arrestin 2 negatively regulates lung cancer progression by inhibiting the TRAF6 signaling axis for NF-κB activation and autophagy induced by TLR3 and TLR4. Cell Death Dis..

[73] Liu Y., Zhou Q., Ye F., Yang C., Jiang H. (2023). Gut microbiota-derived short-chain fatty acids promote prostate cancer progression via inducing cancer cell autophagy and M2 macrophage polarization. Neoplasia.

[74] Schneider A.M., Feehan R.P., Sennett M.L., Wills C.A., Garner C., Cong Z., Billingsley E.M., Flamm A.F., Shantz L.M., Nelson A.M. (2024). TLR3 activation mediates partial epithelial-to-mesenchymal transition in human keratinocytes. Life Sci. Alliance.

[75] Park G.B., Chung Y.H., Kim D. (2017). Induction of galectin-1 by TLR-dependent PI3K activation enhances epithelial-mesenchymal transition of metastatic ovarian cancer cells. Oncol. Rep..

[76] Niu L., Yang W., Zhou W., Duan L., Wang Q., Wang X., Li Y., Xu C., Zhang Y., Liu J. (2025). TGIF2-mediated HMGB3 overexpression promotes esophageal squamous cell carcinoma proliferation and metastasis through TLR3/TGF-β signaling. Genes. Dis..

[77] Youssef K.K., Nieto M.A. (2024). Epithelial-mesenchymal transition in tissue repair and degeneration. Nat. Rev. Mol. Cell Biol..

[78] Shibue T., Weinberg R.A. (2017). EMT, CSCs, and drug resistance: The mechanistic link and clinical implications. Nat. Rev. Clin. Oncol..

[79] Duan H., Noma T., Goel A. (2026). Aronia Berry Extract Inhibits Cancer Stemness and Overcomes 5-Fluorouracil Resistance by Targeting TLR3/NF-κB Signaling in Colorectal Cancer. Pharmaceuticals.

[80] Veyrat M., Durand S., Classe M., Glavan T.M., Oker N., Kapetanakis N.I., Jiang X., Gelin A., Herman P., Casiraghi O. (2016). Stimulation of the toll-like receptor 3 promotes metabolic reprogramming in head and neck carcinoma cells. Oncotarget.

[81] Han S., Xu W., Wang Z., Qi X., Wang Y., Ni Y., Shen H., Hu Q., Han W. (2016). Crosstalk between the HIF-1 and Toll-like receptor/nuclear factor-κB pathways in the oral squamous cell carcinoma microenvironment. Oncotarget.

[82] Matijevic Glavan T., Cipak Gasparovic A., Vérillaud B., Busson P., Pavelic J. (2017). Toll-like receptor 3 stimulation triggers metabolic reprogramming in pharyngeal cancer cell line through Myc, MAPK, and HIF. Mol. Carcinog..

[83] Conforti R., Ma Y., Morel Y., Paturel C., Terme M., Viaud S., Ryffel B., Ferrantini M., Uppaluri R., Schreiber R. (2010). Opposing effects of toll-like receptor (TLR3) signaling in tumors can be therapeutically uncoupled to optimize the anticancer efficacy of TLR3 ligands. Cancer Res..

[84] Lau W.H., Zhu X.G., Ho S.W.T., Chang S.C., Ding J.L. (2017). Combinatorial treatment with polyI:C and anti-IL6 enhances apoptosis and suppresses metastasis of lung cancer cells. Oncotarget.

[85] Singh A., Devkar R., Basu A. (2020). Myeloid Differentiation Primary Response 88-Cyclin D1 Signaling in Breast Cancer Cells Regulates Toll-Like Receptor 3-Mediated Cell Proliferation. Front. Oncol..

[86] Pelka K., Bertheloot D., Reimer E., Phulphagar K., Schmidt S.V., Christ A., Stahl R., Watson N., Miyake K., Hacohen N. (2018). The Chaperone UNC93B1 Regulates Toll-like Receptor Stability Independently of Endosomal TLR Transport. Immunity.

[87] Harris K.G., Coyne C.B. (2015). Unc93b Induces Apoptotic Cell Death and Is Cleaved by Host and Enteroviral Proteases. PLoS ONE.

[88] Palchetti S., Starace D., De Cesaris P., Filippini A., Ziparo E., Riccioli A. (2015). Transfected poly(I:C) activates different dsRNA receptors, leading to apoptosis or immunoadjuvant response in androgen-independent prostate cancer cells. J. Biol. Chem..

[89] Colapicchioni V., Palchetti S., Pozzi D., Marini E.S., Riccioli A., Ziparo E., Papi M., Amenitsch H., Caracciolo G. (2015). Killing cancer cells using nanotechnology: Novel poly(I:C) loaded liposome-silica hybrid nanoparticles. J. Mater. Chem. B.

[90] Hafner A.M., Corthésy B., Textor M., Merkle H.P. (2016). Surface-assembled poly(I:C) on PEGylated PLGA microspheres as vaccine adjuvant: APC activation and bystander cell stimulation. Int. J. Pharm..

[91] Nguyen B.L., Phung C.D., Pham D.V., Le N.D., Jeong J.H., Kim J., Kim J.H., Chang J.H., Jin S.G., Choi H.G. (2023). Liposomal co-delivery of toll-like receptors 3 and 7 agonists induce a hot triple-negative breast cancer immune environment. J. Control Release.

[92] Menendez D., Lowe J.M., Snipe J., Resnick M.A. (2016). Ligand dependent restoration of human TLR3 signaling and death in p53 mutant cells. Oncotarget.

[93] Le Naour J., Kroemer G. (2023). Trial watch: Toll-like receptor ligands in cancer therapy. Oncoimmunology.

[94] Rolfo C., Giovannetti E., Martinez P., McCue S., Naing A. (2023). Applications and clinical trial landscape using Toll-like receptor agonists to reduce the toll of cancer. NPJ Precis. Oncol..

[95] Lacour J., Lacour F., Spira A., Michelson M., Petit J.Y., Delage G., Sarrazin D., Contesso G., Viguier J. (1980). Adjuvant treatment with polyadenylic-polyuridylic acid (Polya.Polyu) in operable breast cancer. Lancet.

[96] Jeung H.C., Moon Y.W., Rha S.Y., Yoo N.C., Roh J.K., Noh S.H., Min J.S., Kim B.S., Chung H.C. (2008). Phase III trial of adjuvant 5-fluorouracil and adriamycin versus 5-fluorouracil, adriamycin, and polyadenylic-polyuridylic acid (poly A:U) for locally advanced gastric cancer after curative surgery: Final results of 15-year follow-up. Ann. Oncol..

[97] Mukherjee S., Boland P.M., Grimm M., Slomba R., Attwood K., Iyer R., Kalinski P. (2022). Abstract CT105: Initial results of a phase II study evaluating a chemokine-modulatory (CKM) regimen in patients with colorectal cancer metastatic to the liver. Cancer Res..

[98] Slingluff C., Mauldin I., Gaughan E., Gaughan E., Dillon P., Opyrchal M., Puzanov I., Kruse M., Gastman B., Friedlander P. (2021). 337 Intratumoral immune therapy for recurrent breast cancer with polyICLC, and tremelimumab combined with systemic durvalumab. J. Immunother. Cancer.

[99] Aznar M.A., Planelles L., Perez-Olivares M., Molina C., Garasa S., Etxeberría I., Perez G., Rodriguez I., Bolaños E., Lopez-Casas P. (2019). Immunotherapeutic effects of intratumoral nanoplexed poly I:C. J. Immunother. Cancer.

[100] Marquez-Rodas I., Dalle S., Castanon E., Sanmamed M.F., Arance A.M., Cerezuela-Fuentes P., Huertas R.M., Rodríguez-Moreno J.F., Gonzalez-Cao M., Muñoz-Couselo E. (2021). Combination of radiomic and biomarker signatures as exploratory objective in a phase II trial with intratumoral BO-112 plus pembrolizumab for advanced melanoma. J. Clin. Oncol..

[101] Rodas I.M., Saiag P., Merino L.d.l.C., de la Cruz Merino L., Dutriaux C., Rodríguez-Moreno J., Robert C., Arance A., Castañón Álvarez E., Cerezuela-Fuentes P. (2021). 961 Preliminary results of a phase 2 study of intratumoral administration of BO-112 with pembrolizumab in patients with advanced melanoma that have progressive disease on anti-PD-1-based therapy. J. Immunother. Cancer.

[102] Le Naour J., Thierry S., Scuderi S.A., Boucard-Jourdin M., Liu P., Bonnin M., Pan Y., Perret C., Zhao L., Mao M. (2023). A Chemically Defined TLR3 Agonist with Anticancer Activity. Oncoimmunology.

